# RXR Gamma Enables Oligodendrocyte Differentiation by Suppressing Sonic Hedgehog Signaling

**DOI:** 10.1002/glia.70151

**Published:** 2026-04-03

**Authors:** Vito Antonio Baldassarro, Quentin Brassart, Valérie Fraulob, Laura Calzà, Wojciech Krezel

**Affiliations:** ^1^ Institut de Génétique et de Biologie Moléculaire et Cellulaire, Centre National de la Recherche Scientifique UMR 7104, Institut national de la santé et de la recherche médicale U 1258, Illkirch, Université de Strasbourg Strasbourg France; ^2^ Department of Veterinary Medical Science University of Bologna Bologna Italy; ^3^ Department of Pharmacy and Biotechnology University of Bologna Bologna Italy

**Keywords:** oligodendrocyte progenitors, oligodendrogenesis, retinoid X receptors, sonic hedgehog, thyroid hormone

## Abstract

Overcoming remyelination failure is one of the main targets in therapeutic strategies for multiple sclerosis. This process requires the differentiation of oligodendrocyte precursor cells (OPCs) to mature myelinating oligodendrocytes (OLs), a process known to be controlled by thyroid hormone, nuclear receptors, and sonic hedgehog (SHH). Retinoid X receptor gamma (RXRg) is one of the nuclear receptors acting as a positive regulator of remyelination, but little is known about its mechanisms of function. Using transcriptomic and pharmacological analysis of primary neural stem cell‐derived OPCs, we show that RXRg is involved in the induction of the thyroid hormone‐driven differentiation process and in refining it toward an oligodendrogenic cell fate. RXRg also emerged as an important negative modulator of SHH expression and signaling, as *Shh* and additional genes from this pathway were found to be strongly upregulated in *Rxrg* ^*−/−*^ OPCs. An inhibition of SHH signaling by cyclopamine or GANT61 entirely normalized the differentiation deficit of *Rxrg* ^*−/−*^ OPCs, but also myelination of newly generated *Rxrg* ^*−/−*^ OLs. Such data indicate a key role of SHH hyperactivity in the oligodendrogenesis block associated with the absence of RXRg. Importantly, hyperactivation of the SHH pathway by purmorphamine or SAG inhibited the oligodendrogenesis and myelination potential of wild‐type OPCs, indicating that SHH hyperactivity can also be a sufficient factor to block OPC differentiation. These results point to RXRg as an important regulator of SHH pathway signaling and underline the need of an optimal, fine‐tuning of SHH signaling to assure successful oligodendrogenesis.

## Graphical abstract

Using pharmacogenetic approaches, Baldassarro et al. demonstrate that RXRγ suppresses the SHH signaling pathway in OPCs to ensure their efficient differentiation following T3 stimulation. Accordingly, *Rxrg* ^*−/−*^ OPCs displayed compromised differentiation which was normalized by the pharmacological inhibition of the hyperactive SHH pathway. These findings establish RXRγ as a pivotal regulator of the balance between OPC proliferation and differentiation and as a new regulator of the SHH signaling pathway.

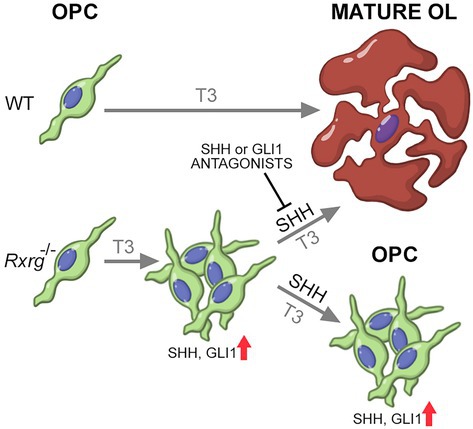

## 1 Introduction

Oligodendrocyte precursor cells (OPCs) derive from multipotent neural stem cells (NSCs) during embryonic and post‐natal central nervous system (CNS) development, during which they are responsible for developmental myelination. These unipotent precursor cells will replicate and migrate, being produced during different spatially and temporally separated waves, three in the brain and two in the spinal cord (Bergles and Richardson 2016). During this process, the generated OPC populations reach the unmyelinated axons, exit the cell cycle, and activate the differentiation machinery, leading to the generation of mature oligodendrocytes. These postmitotic cells wrap the axons with spiral protrusion of their plasma membrane, producing the myelin sheaths (El Waly et al. 2014). Part of the OPCs produced during development remain in an undifferentiated state, colonizing the whole gray and white matter. They account for 5%–8% of the whole cells in the adult CNS, and are recognized by the expression of chondroitin sulfate proteoglycan 4, known as NG2 (neural/glial antigen 2) marker (Dawson et al. 2003).

During adult life, quiescent OPCs are the major cell type overseeing the physiological myelin turnover and myelin repair in pathological conditions. The so‐called “remyelination” process is induced by myelin damage, leading to recruitment of resident OPCs and activating their replication and migration, finally re‐wrapping the demyelinated axons (Levine et al. 2001). Recent studies indicate that NSCs and neuroblasts may also contribute to the remyelination process by differentiating into oligodendrocytes (El Waly et al. 2022).

Both developmental and adult regenerative myelination (remyelination) are orchestrated by a wide range of factors. Paracrine signals, neurotransmitters, growth factors, extracellular matrix components and cell–cell interactions guide the OPCs throughout the whole process, balancing their self‐renewal capacity with the induction and progression of differentiation (Baydyuk et al. 2020). One of the main factors directing this complex machinery is thyroid hormone (TH), more precisely the intracellular level of its active form, triiodothyronine (T3), which is needed to induce cell cycle exit and differentiation of the OPCs (Billon et al. 2001; Calzà et al. 2018). T3 typically acts through a gene regulatory mechanism as a ligand for thyroid hormone receptors (TRa and TRb), belonging to the superfamily of nuclear receptors (NRs). These act as homodimers or heterodimers with retinoid X receptors (RXRα, β, and γ) to regulate the expression of specific genes containing thyroid hormone‐response elements (TREs) (Baldassarro et al. 2021). Beyond its genomic action, T3 also shows non‐genomic activity, which is also fundamental for the regulation of OPC biology (Giammanco et al. 2020; Lee and Petratos 2016). However, all the mediators involved and the downstream effects of such regulatory steps are not entirely understood. In this context, it has been described that the αvβ3 integrin, a truncated form of TRa, could be the main mediator acting through regulation of the sonic hedgehog (SHH) pathway (Emamnejad et al. 2022; Giammanco et al. 2020).

At present it is not clear which aspect of TH activity can be attributed to one of these three modes of TH signaling. Several lines of evidence suggest, however, that signaling through TR‐RXR heterodimers might be particularly relevant for OPC differentiation. All three RXR isotypes (RXRa, b and g), like TRs, act as ligand‐dependent transcription factors and in physiological conditions their activities are modulated by 9‐*cis*‐13,14‐dihydroretioic acid, an endogenous RXR ligand and active form of vitamin A5/X (Krężel et al. 2021). In addition to TRs, RXRs are known to form heterodimers with several NRs, thereby contributing to regulation of multiple signaling pathways frequently in a ligand‐dependent manner (Sharma et al. 2022). All three RXRs play a role in OPC biology, however the RXRg isotype seems to be fundamental for the differentiation process and for remyelination after myelin damage (Dulamea 2017; Huang et al. 2011; Lefebvre et al. 2010). Accordingly, high RXRg expression positively correlates with the differentiation potential of OPCs in active lesions, whereas its absence is characteristic of non‐differentiating OPCs in chronic lesions in multiple sclerosis (Huang et al. 2011). Furthermore, RXRg presence or pharmacological activation promoted OPC differentiation in mouse spinal cord following myelin lesion (Huang et al. 2011) or in cultured NSC‐derived OPCs in vitro (Baldassarro et al. 2019). This latter work revealed a strong differentiation block in *Rxrg* ^*−/−*^ OPCs carrying a null mutation of *Rxrg*, a phenotype resembling the OPC differentiation block observed in multiple sclerosis (MS). MS is the pivot disease in which the remyelination block plays a major role, but the same pathological mechanism is involved in several CNS demyelinating conditions, including diseases and lesions (Baldassarro et al. 2022).

Several studies have implicated the SHH pathway in OPC differentiation, although it appears to have opposing effects depending on the experimental system, which may reflect differences in cell types and CNS region, or perhaps specific time‐points in developmental or regenerative myelination. In general, SHH signaling is recognized as a positive regulator of oligodendrogenesis both during development and adult life (Wang and Almazan 2016). During development, OPCs are induced from the ventral neural tube by SHH produced in ventral midline structures (Fuccillo et al. 2006), while Gli2 and Gli3 seem to regulate oligodendrocyte development in the ventral spinal cord (Tan et al. 2006). Also in the adult brain, SHH was described to promote oligodendrocyte generation if acting in OPCs (Emamnejad et al. 2022; Loulier et al. 2006). Opposite effects were however reported. Several studies documented promotion of OL maturation and remyelination following inhibition of the SHH pathway, especially in the context of neuroblasts or NSC‐derived OPCs (Namchaiw et al. 2019; Radecki et al. 2020; Samanta et al. 2015). The mechanisms regulating the equilibrium between activation/inhibition of the SHH pathway in these studies remain unclear.

To investigate the mechanisms of RXRg control of OPC differentiation and maturation, we used NSC‐derived OPCs isolated from *Rxrg* ^*−/−*^ mice. Using transcriptomic analysis, we identified hyperactivity of the SHH signaling pathway as significantly associated with differentiation impairment in *Rxrg* ^*−/−*^ OPCs. The involvement of such hyperactivity in OPC differentiation block was documented by a pharmacological approach: indeed, pharmacological downregulation of the SHH pathway was able to prevent the differentiation and maturation deficit observed in *Rxrg* ^*−/−*^ OPCs and OLs, respectively. We also showed that overactivation of SHH in wild‐type (WT) OPCs leads to a differentiation and maturation block in a dose‐dependent manner. Our data implicate RXRg as an important regulator of the SHH signaling pathway, and highlight the potential of inhibition of the SHH pathway as a strategy for pharmacological intervention to overcome the differentiation block in OPCs lacking RXRg expression.

## 2 Materials and Methods

### 2.1 Neural Stem Cell‐Derived OPC Cultures and Treatments

The experiments involving animals described herein were carried out according to the European Community Council Directives (86/609/EEC) and complied with the guidelines published in the NIH Guide for the Care and Use of Laboratory Animals. The WT and *Rxrg* ^*−/−*^ mice used in this study were raised on mixed C57BL/6J x 129SVpass genetic background (Krezel et al. 1996). To obtain NSC‐derived OPCs, NSCs were isolated from E13.5 fetal forebrain and induced for OL lineage differentiation as described (Baldassarro 2021) (Figure S2). Briefly, tissue was mechanically dissociated and incubated for 15 min in non‐enzymatic dissociation buffer (Sigma‐Aldrich, Ref. C‐5914) at 37°C with gentle shaking. Dissociated cells were resuspended in serum‐free medium (DMEM/F12 GlutaMAX; 8 mmol/L HEPES; 100 U/100 μg Penicillin/Streptomycin; 1 × delipidated B27; 1 × N2; 10 ng/mL bFGF; 10 ng/mL EGF). Cells were plated in suspension at a density of 10 cells/μL. Half of the medium was changed every 3 days. Neurospheres were allowed to proliferate until they attained a diameter of approximately 100 μm. To obtain oligospheres, the primary neurospheres were centrifuged, the pellet was mechanically dissociated by pipetting, and the cells were counted and plated again at a density of 10 cells/μL in OPC medium (DMEM/F12 GlutaMAX; 8 mmol/L HEPES; 100 U/100 μg Penicillin/Streptomycin; 1 × B27; 1 × N2; 20 ng/mL bFGF; 20 ng/mL PDGF). After 3 days of culture, the oligospheres were centrifuged and the pellet was mechanically dissociated to obtain a single cell suspension. After cell counting, the cells were plated at a density of 3000 cells/cm^2^ on poly‐D,L‐ornithine (50 μg/mL)/laminin (5 μg/mL) coated plates, in OPC medium.

To induce OL differentiation and maturation, after 3 days in vitro (DIVs), the OPC medium was replaced with OL differentiation medium (DMEM/F12 GlutaMAX; 8 mmol/L HEPES; 100 U/100 μg Penicillin/Streptomycin; 1 × B27; 1 × N2; 50 nM T3; 10 ng/mL CNTF; 1 × *N*‐acetyl‐L‐cysteine‐NAC).

Treatments with SHH pathway inhibitors were performed at the same time as the differentiation induction by T3 exposure by addition of the Gli1 inhibitor GANT61 (10 μM; Sigma‐Aldrich), or the Smo inhibitor cyclopamine (10 μM; Sigma‐Aldrich). After 3 days the inhibitors were removed, and cells were cultured in standard differentiation medium for further 3 days. Using the same protocol, two different SHH agonists were also tested, that is, SAG (1.5 μM; MedChemExpress) and purmorphamine (1 μM; MedChemExpress) (Figure S2).

### 2.2 Post‐Natal Primary OPC Cultures

Mouse pups at P7 were sacrificed by decapitation. Mouse brains were isolated in HBSS (minus calcium and magnesium) and meninges were removed. The two brain lobes were then isolated and put in a C‐tube (Miltenyi, Cat N. 130‐093‐237). Pre‐heated dissociation mix containing papain (46 mg/mL; Worthington, Cat. N. WOLS03126), DNAse 1% (Worthington, Cat. N. WOLS02139), and 12.4 mg/mL L‐cysteine (Sigma‐Aldrich, Cat. N. C7880‐100 g) was added and brains were gently dissociated for 2 min using a MACS dissociator (Miltenyi, 130‐093‐235). The resulting homogenous cell suspension was then filtered using a 70 μm filter. The cell suspension was washed with HBSS (containing calcium and magnesium) and then centrifuged at 4°C at 300 *g* for 5 min. The supernatant was removed. The operation was repeated three times. Cells were finally incubated in sorting buffer (PBS + BSA 0.5%) for 5 min, and centrifuged at 300 g at 4°C for 5 min.

Cells were resuspended in 80 μL of sorting buffer and 10 μL of blocking solution from PDGFRa Microbead kit (Miltenyi, Cat. N. 130‐101‐547). Gentle mixing by pipetting was performed and cells were incubated at 4°C for 10 min. Then 10 μL of PDGFRa beads were added to the solution, gently mixed, and incubated for 15 min at 4°C. Cell suspension was finally centrifuged for 5 min at 300 g at 4°C, and resuspended in 1 mL of sorting buffer.

The miniMACS separator kit was used to filter OPCs (Miltenyi, cat. N. 130‐090‐312). MS columns (Miltenyi, cat. N. 130‐042‐201) were equilibrated using 500 μL of sorting buffer. Then the cell suspension was added to the column, followed by 3 washes with 500 μL of sorting buffer. Cells were then flushed using 1 mL of pre‐heated OPC cell medium, counted, and seeded at 25,000 cells per 24‐well plate.

Cells were cultured as described previously for NSC‐derived OPCs, that is, for 3 days using proliferation medium, and then 3 days in differentiation medium containing T3.

### 2.3 Immunocytochemistry

Indirect immunofluorescence was used to identify OPCs (NG2 and PDGFRα), mature OLs (MBP), replicating cells (Ki67), and SHH expression. Primary antibodies were used as follows: rabbit anti‐NG2 (gift from Dr. Bill Stallcup), rat anti‐PDGFRα (BD Pharmingen), chicken anti‐MBP (Aveslab), rabbit anti‐Ki67 (Synaptic Systems), rabbit anti‐SHH (clone C9C5; Cell Signaling Technologies) (1/200). Cells were fixed in 4% PFA for 10 min, blocked and permeabilized using PBS‐0.1% Triton + BSA 5%, and incubated with respective primary antibodies as indicated in the Results section. Secondary antibodies were used as follows: goat anti‐chicken coupled to Alexa Fluor 488 (Invitrogen‐A11039), donkey anti‐rabbit coupled to Alexa Fluor 488 (Invitrogen‐A21206), donkey anti‐rabbit coupled to Alexa Fluor 555 (Invitrogen‐A31572) and goat anti‐rat coupled to Alexa Fluor 647 (Invitrogen‐A21247). During the secondary antibody incubation, cells were also stained with DAPI for nuclear staining. Finally, the cells were washed in PBS and mounted in Aqua‐Poly/mount (Polysciences).

### 2.4 Confocal Microscopy and Image Analysis

Confocal microscopy was used to acquire images for analysis of differentiation and maturation of NSC‐derived OPCs. Images were acquired with a Nikon Ti‐E fluorescence microscope, connected to an A1R confocal system (Nikon, Minato, Tokyo, Japan) consisting of a series of diode lasers with an output wavelength of 405 nm, an air‐cooled argon‐ion laser system with 488 nm output, and a yellow diode‐pump solid‐state laser system with a 561 nm wavelength output. Images were acquired using a 40 × lens with 1024 × 1024 resolution, and all z‐stacks were collected in compliance with optical section separation (z‐interval) values suggested by the NIS‐Elements AR 3.2 software (1 μm).

For the differentiation analysis, the maximum intensity projections of whole cells were used and for each field the total number of nuclei stained with DAPI was counted as the total cell number. The specific markers were used to count the marker‐positive cells, and the percentage was calculated based on whole cell number per field.

For the maturation analysis, the IMARIS software (v. 9.72) was used on 3D confocal images. Isosurfaces were produced from single mature MBP‐positive OLs and were used to measure the whole volume for each cell and cell morphology using the Sholl analysis algorithm.

For all analyses, a minimum number of five fields per coverslip in three independent replicates was used.

### 2.5 Transcriptomic Analysis

To investigate transcriptional changes associated with RXRg deletion during differentiation or in non‐differentiating conditions, high‐throughput RNA sequencing was performed on samples obtained from WT and *Rxrg* ^*−/−*^ OPCs at 24 h after T3 or vehicle exposure. Total RNA was isolated from approximately 10 million OPCs obtained from distinct primary cultures (*n* = 4 samples/genotype/treatment) using RNeasy Micro kits (Qiagen) following the manufacturer's instructions.

Total RNA‐Seq libraries were generated from 165 to 300 ng of total RNA using TruSeq Stranded Total RNA Library Prep Gold kit and TruSeq RNA Single Indexes kits A and B (Illumina, San Diego, CA), according to the manufacturer's instructions. Briefly, cytoplasmic and mitochondrial ribosomal RNA (rRNA) was removed using biotinylated, target‐specific oligonucleotides combined with Ribo‐Zero rRNA removal beads. Following purification, the depleted RNA was fragmented into small pieces using divalent cations at 94°C for 8 min. Cleaved RNA fragments were then copied into first strand cDNA using reverse transcriptase and random primers, followed by second strand cDNA synthesis using DNA polymerase I and RNase H. Strand specificity was achieved by replacing dTTP with dUTP during second strand synthesis. The double stranded cDNA fragments were blunted using T4 DNA polymerase, Klenow DNA polymerase and T4 polynucleotide kinase. A single “A” nucleotide was added to the 3′ ends of the blunt DNA fragments using a Klenow fragment (3′ to 5′ exo minus) enzyme. The cDNA fragments were ligated to double stranded adapters using T4 DNA ligase. The ligated products were enriched by PCR amplification (30 s at 98°C; [10 s at 98°C, 30 s at 60°C, 30 s at 72°C] × 12 cycles; 5 min at 72°C). Surplus PCR primers were further removed by purification using AMPure XP beads (Beckman–Coulter, Villepinte, France), and the final cDNA libraries were checked for quality and quantified using capillary electrophoresis. All the libraries were sequenced on an Illumina HiSeq 4000 sequencer as single‐read 50 base reads. Image analysis and base calling were carried out using RTA v.2.7.3 and bcl2fastq v.2.17.1.14.

Reads were preprocessed using Cutadapt v.1.10 (Martin 2011) in order to remove adaptors and low‐quality sequences and reads shorter than 40 bp. rRNA sequences were removed for further analysis. Reads were mapped onto the mm10 assembly of the *Mus musculus* genome using STAR v.2.5.3a (Dobin et al. 2013). Gene expression was quantified from uniquely aligned reads using HTSeq‐count v.0.6.1p1 (Anders et al. 2015) with annotations from Ensembl release 98 and union mode. Only non‐ambiguously assigned reads were retained for further analyses. Comparisons of interest were performed using R 3.3.2 with DEseq2 version 1.16.1 (Love et al. 2014). More precisely, read counts were normalized from the estimated size factors using the median‐of‐ratios method, and a Wald test was used to estimate the *p*‐values. Batch effect resulting from the time of RNA extraction was considered in the statistical model (design = ~batch + condition in the DESeqDataSetFromMatrix function). *p*‐values were then adjusted for multiple testing with the Benjamini and Hochberg method (Benjamini and Hochberg 1995).

Gene set and pathway analyses were performed using GSEA (Subramanian et al. 2005) and iDEP (Ge et al. 2018) and associated pathway databases as indicated in the text. To assess T3‐dependent transcriptional changes associated with OPC differentiation, we identified those deregulated genes/transcripts (DEGs) which carry TRa or TRb binding‐site(s) (Zekri et al. 2022), and thus may act as potential direct TR target genes.

### 2.6 qPCR Analysis

Total RNA was isolated from cultured primary post‐natal OPCs using RNeasy Micro kits, with concurrent genomic DNA removal. cDNA was synthesized from 1 μg RNA using QuantiTect Reverse Transcription Kit (Qiagen) according to the manufacturer's recommendations.

qPCR reactions were performed using cDNA in triplicates in a LightCycler 480 (Roche) using DNA SYBR Green I Master kit (Roche). Relative quantification of mRNA was obtained using the comparative cycle threshold (Cq) method. Cq values were obtained for each sample and normalized for the housekeeping gene *36b4* to account for input quantity. Specific primers were used to analyze the expression of the housekeeping gene and the target gene *Shh* (Table 1), with a tested efficiency approximately to 2. The formula 2^−(ΔΔCq)^ was used as a semi‐quantification method, initially normalizing the Cq of the target gene with the housekeeping gene (ΔCq), and subsequently with a control group (ΔΔCq). The relative expression is then given as fold change relative to the WT group.

**TABLE 1:** Primer sequences.

Gene	Forward	Reverse
*Shh*	AAGCTGACCCCTTTAGCCTA	TCGGAGTTTCTTGTGATCTTCC
*36b4*	ACCCTGAAGTGCTCGACATC	AGGAAGGCCTTGACCTTTTC

### 2.7 Statistical Analysis

Data are reported as mean ± SD. The Prism software (v.10; GraphPad Software, San Diego, CA, USA) was used for statistical analyses and graph generation. Differentiation experiments to assess OPC differentiation/maturation in WT and *Rxrg* ^*−/−*^ NSC‐derived OPC cultures and the effects of pharmacological treatments were replicated by two independent operators. Data were collected from at least three independent experiments and analyzed by two‐way ANOVA with genotype and treatment as independent factors followed by Dunnett's post hoc test, or student's *t*‐test and one‐way ANOVA for two group comparison or treatment effect with genotype as a single independent variable, as indicated in the figure legends. The results were considered significant when the probability of their occurrence as a result of chance alone was below 5% (*p* < 0.05).

## 3 Results

### 3.1 Deletion of Rxrg Compromises Only Partially T3‐Dependent Transcriptional Programs in Rxrg^−/−^ OPCs

**FIGURE 1:**
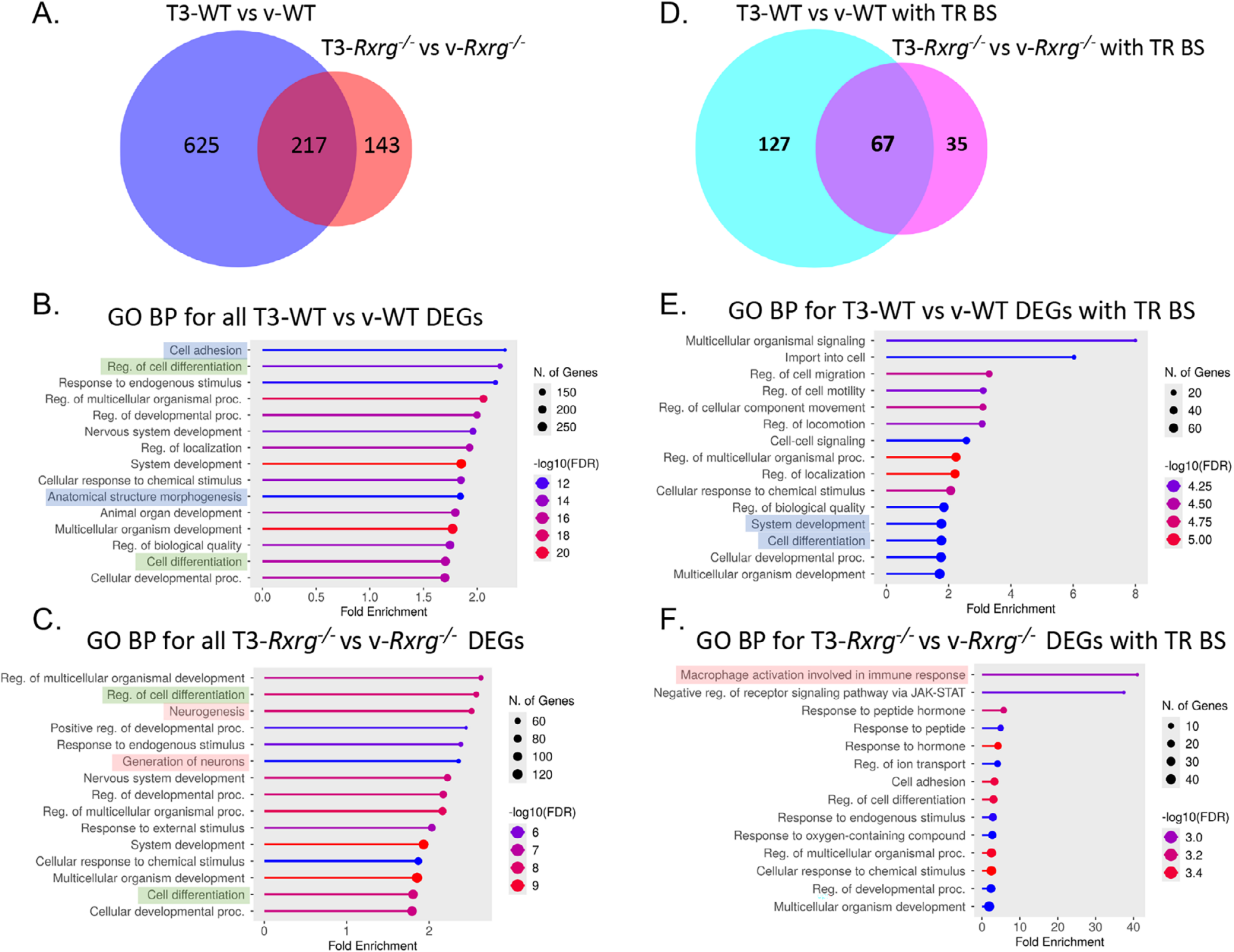
RXRg conditions thyroid hormone responses of NSC‐derived OPCs in vitro. (A–C) Venn diagram representation of T3‐induced transcriptional changes revealed by RNAseq in WT and *Rxrg* ^*−/−*^ OPCs at 24 h after treatment (A) and ShinyGO v0.81 analyses of GO BP in T3‐treated WT (B) and *Rxrg* ^*−/−*^ OPCs (C). (D–F) T3‐dependent regulation of genes with thyroid hormone receptor‐binding sites (TR BS) in WT and *Rxrg* ^*−/−*^ OPCs at 24 h after treatment (D) and corresponding ShinyGO analyses of GO BP in T3‐treated WT (E) and *Rxrg* ^*−/−*^ OPCs (F). Selected biological processes modulated by T3 in both genotypes were highlighted in green, whereas WT‐specific changes are in blue and *Rxrg* ^*−/−*^‐specific changes in pink. DEGs, differentially expressed genes; GO BP, gene ontology biological processes; T3‐WT/T3‐*Rxrg* ^*−/−*^, Wild‐type (WT)/*Rxrg* ^*−/−*^ OPC cultures treated with triiodothyronine (T3); TR BS, thyroid hormone receptor‐binding site; v‐WT/v‐*Rxrg* ^*−/−*^, Wild‐type (WT)/*Rxrg* ^*−/−*^ OPC cultures treated with vehicle.

To investigate the mechanisms of compromised T3‐dependent OPC differentiation in *Rxrg* ^*−/−*^ cultures, we first asked whether and to what extent deletion of *Rxrg* abrogates molecular response to thyroid hormone. To this end, we compared transcriptional changes induced by T3 in WT and *Rxrg* ^*−/−*^ OPCs at 24 h after addition of T3 (50 nM) as compared to vehicle treatment, used as control for non‐differentiating condition. Heatmap overview of transcriptomic data revealed stark differences between WT vs. *Rxrg* ^*−/−*^ and vehicle vs. T3‐treated cells (Figure S1). We identified 842 transcripts (corresponding to 712 annotated genes) as significantly changed (adjusted *p*‐value < 0.05 and Log2 FC > 1) by T3 treatment in WT OPCs, as opposed to only 360 transcripts (292 genes) in *Rxrg* ^*−/−*^ OPCs (a complete list of transcripts is accessible in Supporting Information). A vast majority of these transcripts were upregulated following T3 treatment in both WT and *Rxrg* ^*−/−*^ OPCs, although in comparison to 517 transcripts in WT only 282 were upregulated in *Rxrg* ^*−/−*^ OPCs. Globally, out of 842 transcripts affected (up‐ and down‐regulated) by T3 treatment in WT cells, only 217 were shared with transcripts regulated by T3 in *Rxrg* ^*−/−*^ cells, whereas 625 transcripts were affected by T3 exclusively in WT cells indicating that in absence of *Rxrg* their control by T3 is abrogated (Figure 1A). A smaller fraction of 143 genes acquired abnormal responsiveness to T3 exclusively in *Rxrg* ^*−/−*^ as compared to WT OPCs, which means that they changed their expression levels following T3 treatment in *Rxrg* ^*−/−*^ but not WT OPCs (Figure 1A).

Functional annotation of T3‐dependent genes in WT and *Rxrg* ^*−/−*^ OPCs highlighted regulation of several common biological processes including, in particular, cellular differentiation, although with some important genotype‐dependent differences (Figure 1B and Table S1 Reference source not found. “GO BP_T3WT vs. vWT”). Thus, in addition to a lower number of genes related to *regulation of cell differentiation* in the absence of *Rxrg* (117 genes in WT against 58 genes in *Rxrg* ^*−/−*^ OPCs), several genes were found to be involved in different aspects of developmental processes depending on the genotype (Table S1, GO BP_T3WT vs. vWT, GO_BP_T3Xg vs. vXg). For example, upregulation of *Dio3* illustrating a feedback response to T3 treatment or *Opalin* and *Nkx6.2* indicating partial activation of oligodendrocyte differentiation were observed in both genotypes. However, T3 clearly suppressed several developmental genes exclusively in WT OPCs (*Dll3, Gdf10, Tal1, Draxin, Mycn, Myocd, Slit1*, *Tfap2b*, *Spint1, Areg, Agt, Hoxa7, Inhba, Disp3, Trpc5, Adra2c, Adra2b, Dlx2, Dlx1, Chd5, Ikzf3, Trpv2, Appl2, Nfatc4, Ptprc, Pax8, Shox2, Hsf4, Htr2a, Prtg, Gpr68, Ranbp3l, Vstm2a*), among which some are well known to be involved in neuronal differentiation and/or were identified as negative regulators of oligodendrocyte differentiation and myelination (Achim et al. 2013; Deboux et al. 2020; Li et al. 2015; Petryniak et al. 2007; Wei et al. 2023). Exclusive to T3‐treated WT cells was also induction of a number of developmental genes (*Slc45a3, Tmem182, Il23a, Hspb1, Gjc2, Wnt4, Tnfsf11, Fas, Dct, S1pr5, S1pr3, Tnfrsf12a, Wnt3, Cd109, Dock5, Zfp536, Pkp2, Cxcl14, Grm5, Ripk2, Mbnl3, Gcnt2, Cntn4, Klf5, Tmem98, Scin, Tent5c, Hopx, Zfp36, Grb14, Reck, Ajap1, Metrn, Igf2, Chd3, Loxl2, Nppc, Vim, Itgb3*) (Table S1 GO BP_T3WT vs. vWT, reg. of developmental proc.), out of which *Zif536* is known to be a key factor for induction of oligodendrocyte fate (Dugas et al. 2006; Yang et al. 2013). Cell adhesion or anatomical structure morphogenesis gene sets were observed exclusively in T3‐treated WT OPCs (Figure 1B and Table S1, **GO BP_T3WT vs. vWT**), whereas *neurogenesis* and *generation of neurons* were associated only with T3‐treated *Rxrg* ^*−/−*^ OPCs (Figure 1C and Table S1, **GO BP_T3Xg vs. vXg**). Emergence of this latter functional group of genes in T3‐treated *Rxrg* ^*−/−*^ OPCs (*Sema4d, Tnn, Lhx6, Ngf, Sema3c, Plxnd1, Wnt5b, Ephb1, Rac2, Lgi4, En2, Sema3d, Daam2, Tmem108, Helt, Gap43, Neurod4, Cntn2, Map6, Tafa1, Irx1, Sema3e, Socs2, Chl1, Cck, P2ry12, Atp1b2, Sdk2, Pou4f1, Vwc2, Gper1, Kndc1, Gfap, Gdnf, Ehd1, Bhlhe22, Mobp, Mag, Tgm2, Grin3a, Gldn, Grm7, Stat3, Tspan2, Fstl4, Nkx6‐2, Tppp, Gpr37l1, Trpc4, Ugt8a, Kcna1, Sorl1, Opalin, Eml1, Dio3, Epor, Rgs6, Cpne6, Trf, Mbp*) (Table S1, **GO BP_T3Xg vs. vXg, neurogenesis**) may suggest that RXRg acts as a suppressor of neurogenesis in OPCs under T3 treatment.

To assess whether reduced response to T3 reflects compromised regulation of direct transcriptional targets of TR in *Rxrg* ^*−/−*^ OPCs, we identified among T3‐regulated transcripts potential direct targets defined as genes harboring TR‐binding sites (BS) in their vicinity (±20 kb) and known to be expressed in the striatum and postnatal (P15) mouse brain (Zekri et al. 2022). Among 194 genes carrying TR BS and regulated by T3 in WT OPCs, only 67 (65 upregulated and 2 downregulated) were shared as T3‐regulated genes in *Rxrg* ^*−/−*^ OPCs, whereas 127 DEGs (77 upregulated and 50 downregulated) were observed only in T3‐treated WT cells, indicating that deletion of *Rxrg* abrogated more than 50% of T3‐dependent transcriptional regulation (Figure 1D). Interestingly, following T3 treatment, 35 genes bearing TR BS changed their expression exclusively in *Rxrg* ^*−/−*^ OPCs and a vast majority of these genes (32) were upregulated, suggesting that RXRg may contribute to active repression of some T3‐dependent genes. Globally, these data indicate an approximately 50% reduction of responsiveness to T3 treatment in *Rxrg* ^*−/−*^ OPCs.

Functional annotation of T3‐dependent DEGs carrying TR BS pointed to system development and cell differentiation as biological processes appearing in WT, but not *Rxrg* ^*−/−*^ OPCs (Figure 1D,E and Table S2). This further indicates that T3 treatment was not sufficient to engage *Rxrg* ^*−/−*^ OPCs in the differentiation process, but rather activated a gene expression program typical for macrophage activation (*Syk, Dysf, Sbno2, Sucnr1*) (Figure 1F and Table S2, **GO BP_allT3Xg + TRBS_StrP15**).

These data indicate that the transcriptional responses to T3 which are lost in *Rxrg* ^*−/−*^ OPCs correspond, at least partially, to induction of oligodendrogenesis and suppression of neurogenic (and other cell‐type) differentiation programs, whereas T3‐responses observed exclusively in *Rxrg* ^*−/−*^ OPCs were consistent with loss of oligodendrogenic cell fate on benefit of a neurogenic cell fate.

### 3.2 Rxrg^−/−^ NSC‐Derived OPCs Show a Downregulation of Differentiation‐Related Genes and an Upregulation of the SHH Pathway

As mentioned above, loss of *Rxrg* function only partially compromised T3‐dependent gene transcription, including induction of several oligodendrogenesis‐related genes such as *Opalin, Nkx6.2, Mbp, Mog*, or *Mag*.

**FIGURE 2:**
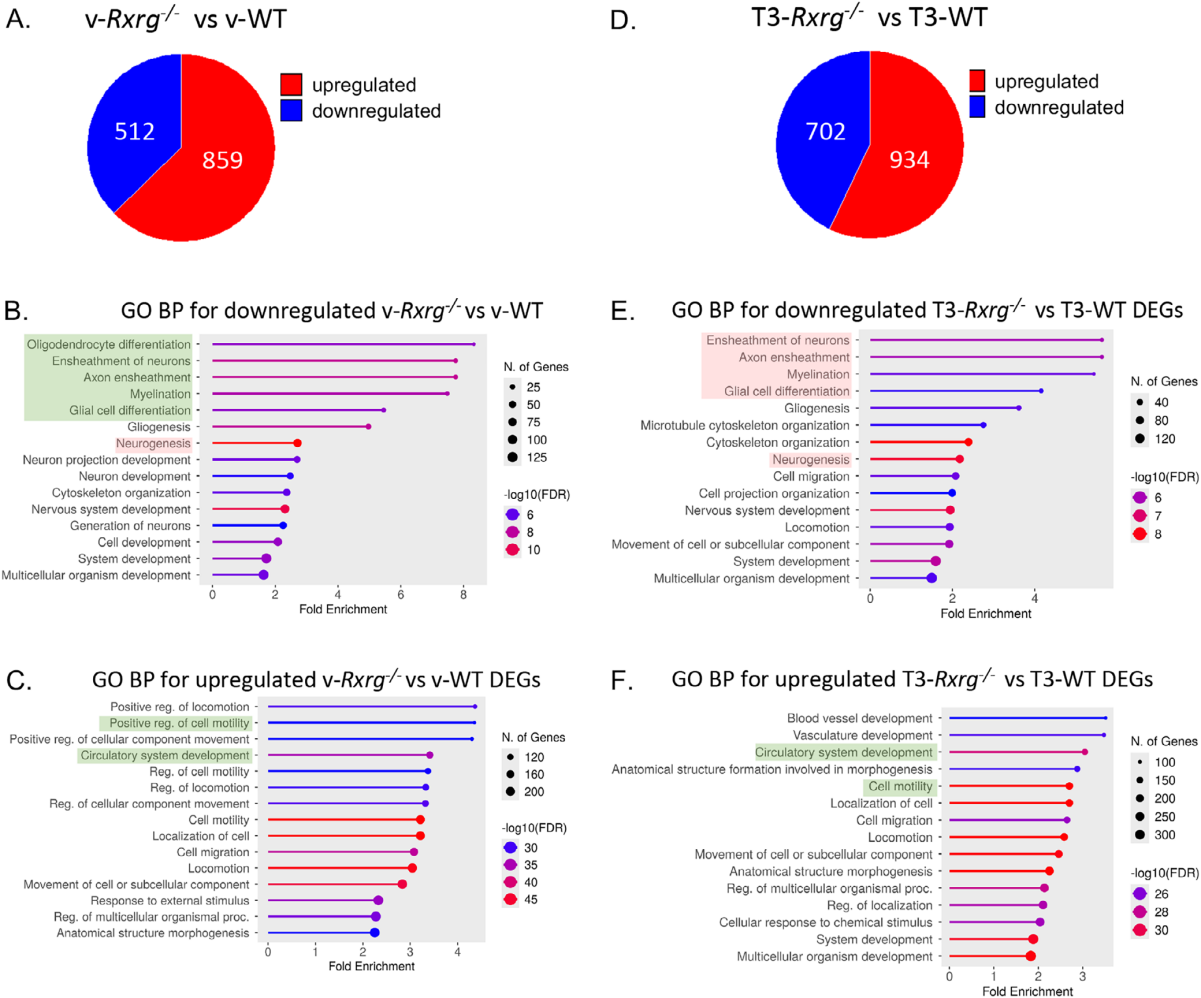
Transcriptional changes associated with loss of RXRg function. (A) Pie chart representing up‐ and downregulated genes in *Rxrg* ^*−/−*^ NSC‐derived OPCs in control conditions of vehicle treatment, and associated GO BP analyses of annotated downregulated (B) and upregulated genes (C). (D) Pie chart illustrating up‐ and downregulated genes in *Rxrg* ^*−/−*^ NSC‐derived OPCs following T3 treatment in comparison with T3‐treated WT OPCs and associated GO BP analyses of annotated downregulated (E) and upregulated genes (F). Selected biological processes modulated by T3 in both genotypes were highlighted in green, whereas WT‐specific changes in blue and *Rxrg* ^*−/−*^‐specific changes are in pink. DEGs, differentially expressed genes; GO BP, gene ontology biological processes; T3‐WT/T3‐*Rxrg* ^*−/−*^, Wild‐type (WT)/*Rxrg* ^*−/−*^ OPC cultures treated with triiodothyronine (T3); TR BS, thyroid hormone receptor binding site; v‐WT/v‐*Rxrg* ^*−/−*^, Wild‐type (WT)/*Rxrg* ^*−/−*^ OPC cultures treated with vehicle.

This suggests that failure of OPC differentiation and myelination reflects not only compromised T3 response, but also other gene deregulations in *Rxrg* ^*−/−*^ OPCs even prior to T3 treatment. To address this possibility, we have investigated DEGs associated with the loss of *Rxrg* function. Among 1371 transcripts (corresponding to 1141 DEGs), 859 were upregulated (789 DEGs) and 512 (351 DEGs) were downregulated (Figure 2A). Gene annotation analyses revealed that *oligodendrocyte differentiation* was the main biological function affected by downregulated genes in *Rxrg* ^*−/−*^ OPCs, reflecting the significant decrease of *Plp1, Myrf, Mobp, Lpar1, Dusp10, Mag, Nkx6‐2, Cnp, Tppp, Mal, Tspan2, Tmem98, Dusp15, Opalin*, and *Sirt2* (Figure 2B and Table S1, **GO BP_down vRxrgKO vs. vWT**). These and additional genes associated with such GO terms as *ensheathment of neurons, myelination, glial cell differentiation* were downregulated in *Rxrg* ^*−/−*^ OPCs (Figure 2B and Table S1, **GO BP_down vRxrgKO vs. vWT**). Likewise, compromised *neurogenesis* reflected reduced expression of 76 genes in *Rxrg* ^*−/−*^ OPCs (Figure 2B and Table S1, **GO BP_down vRxrgKO vs. vWT**). In contrast, analyses of upregulated genes revealed significant enrichment of *cell motility* and *circulatory system development* GO biological processes with a number of genes related to hematoendothelial cell lineage, like *CD34* or *KDR*, suggesting a partial cell fate change of *Rxrg* ^*−/−*^ OPCs (Figure 2C and Table S1, **GO BP_up vRxrgKO vs. vWT**). Strongly represented within these GO BPs were extracellular matrix (ECM)‐related genes, all significantly upregulated in *Rxrg* ^*−/−*^ OPCs (vRxrg vs. vWT, for example, ECM‐related upregulated genes, *Col3a1, Col1a1, Col8a1, Col1a2, Col22a1, Mmp2, Lamb1, Mme, Col18a1, Pcolce, Col6a2, Col4a1, Col4a2, Lamc3, Col6a1, Col5a1, Fndc3c1, Col5a2*), as well as integrin clusters (vRxrg vs. vWT upregulated integrin genes, *Itgb2, Itga3, Itga11, Itgbl1, Itga1*) (Table S1). Strikingly, sonic hedgehog (*Shh*) and its transcriptional target *Gli1* appeared overexpressed and common to different biological processes that were upregulated, at the expense of reduced *oligodendrogenesis* and *myelination*.

### 3.3 Downregulation of Differentiation‐Related Genes and Upregulation of the SHH Pathway in Rxrg^−/−^ NSC‐Derived OPCs Is Maintained After T3 Differentiation Induction

Thus, we found that the OPCs derived from *Rxrg* ^*−/−*^ NSCs at basal, undifferentiated conditions display reduced expression of genes related to cellular differentiation in parallel to about a 10‐fold increase of SHH pathway genes (6.5 and 11‐fold increase of *Shh* and *Gli1*, respectively). These changes could underlie the compromised response of *Rxrg* ^*−/−*^ OPCs to the differentiating stimulus of T3, or be merely a functionally non‐relevant correlate of *Rxrg* deletion prone to normalization after T3 treatment. To address this question, we used RNAseq data to compare *Rxrg* ^*−/−*^ and WT OPCs following T3 treatment (Figure 2D–F). For a total of 1636 significantly altered transcripts (1333 genes), 934 (850 genes) were upregulated and 702 (483 genes) downregulated (Figure 2D and Table S1, **T3RxrgKO vs. T3WT**). Functional annotations of these DEGs did not change significantly with respect to vehicle‐treated *Rxrg* ^*−/−*^ and WT OPCs, and among the top downregulated biological processes, we found ensheathment of neurons, myelination, glial cell differentiation, or neurogenesis, consistent with a failure of OPC differentiation (Figure 2E and Table S1, **GO BP_down T3RxrgKO vs. T3WT**), whereas among the top upregulated processes appeared *cell motility* and *circulatory system development* (Figure 2F and Table S1, **GO BP_up T3RxrgKO vs. T3WT**). Importantly, *Shh* expression as well as expression of its transcriptional effector, *Gli1*, remained highly upregulated in *Rxrg* ^*−/−*^ OPCs (4.5‐ and 10.5‐fold increase with respect to T3‐treated WT OPCs) (Table S1).

### 3.4 Hyperactivity of SHH Is a Key Determinant of Differentiation Block in Rxrg^−/−^ NSC‐Derived OPCs

**FIGURE 3:**
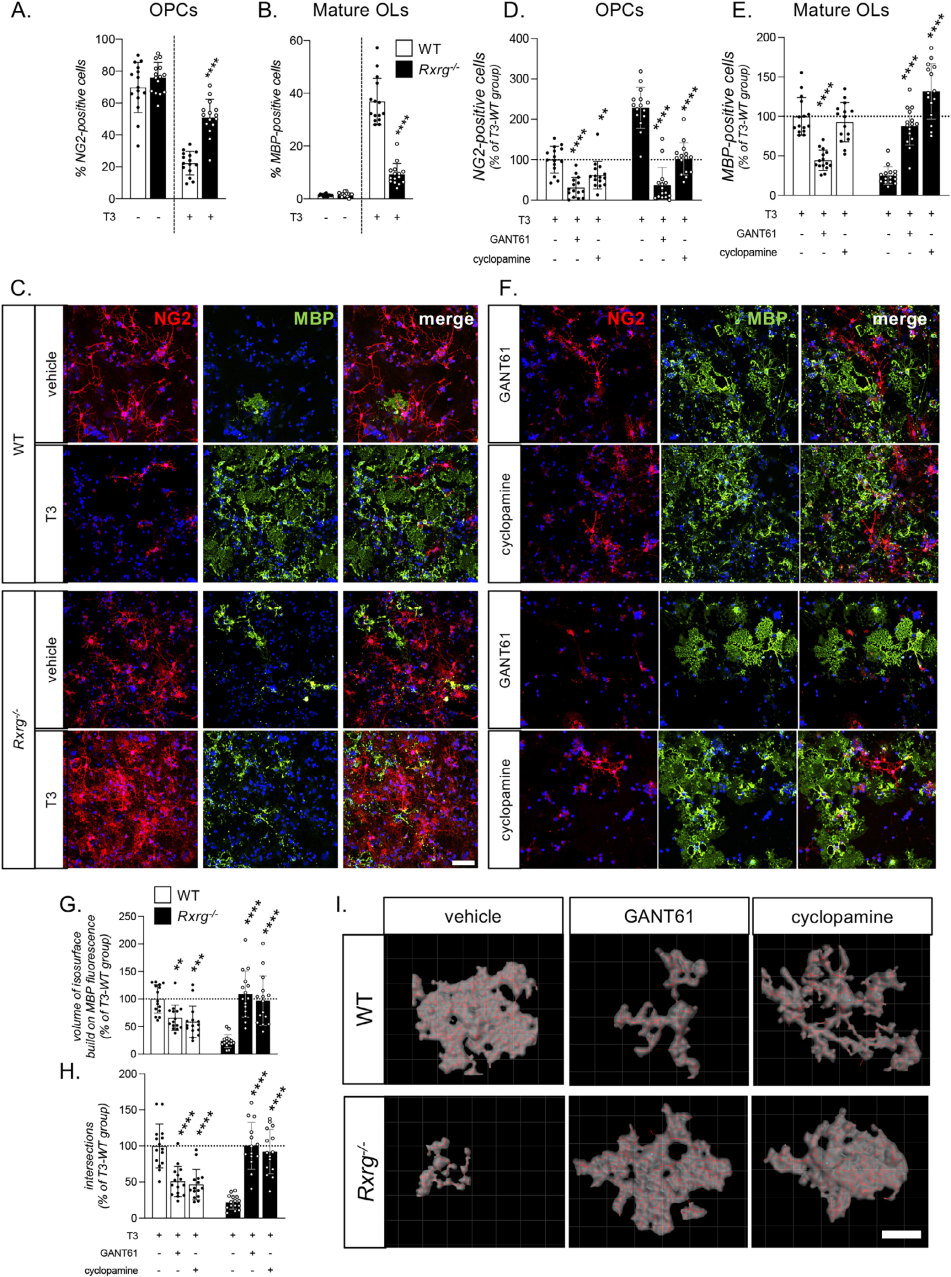
OPC differentiation and maturation are impaired in *Rxrg* ^*−/−*^ cultures through a *Shh*‐dependent mechanism. (A, B) Graphs show the percentage of OPCs (A, NG2‐positive cells) and mature oligodendrocytes (B, MBP‐positive cells) in WT and *Rxrg* ^*−/−*^ cultures at 6 DIVs after exposure to T3 or vehicle. (C) Representative pictures of OPCs (NG2‐positive cells) and mature oligodendrocytes (MBP‐positive cells) in WT and *Rxrg* ^*−/−*^ cultures exposed to T3 or vehicle. Scale bar: 25 μm. (D, E) Graphs show the percentage of OPCs (D, NG2‐positive cells) and mature oligodendrocytes (E, MBP‐positive cells) in WT and *Rxrg* ^*−/−*^ cultures at 6 DIVs after T3 exposure, treated for 3 DIVs with vehicle, GANT61 or cyclopamine. Data are shown as percentage of the WT group treated with vehicle (horizontal dotted line, 100%). (F) Representative picture of OPCs (and mature oligodendrocytes) in WT and *Rxrg* ^*−/−*^ cultures treated with GANT61 or cyclopamine. Scale bar: 25 μm. (G, H) Graphs show the MBP fluorescence‐based isosurface volume (G) and Sholl analysis (H) in WT and *Rxrg* ^*−/−*^ cultures at 6 DIVs after T3 exposure, treated for 3 DIVs with vehicle, GANT61 or cyclopamine. Data are shown as percentage of the WT group treated with vehicle (horizontal dotted line, 100%). (I) Representative pictures of WT and *Rxrg* ^*−/−*^ MBP‐positive cells treated with vehicle, GANT61 or cyclopamine; images were generated with the IMARIS software to show the isosurface (light gray) and filaments (red). Scale bar: 30 μm. Statistical analyses: Student's *t*‐test (A, B), asterisks represent differences between WT and *Rxrg* ^*−/−*^ in the same culture conditions (****p < 0.0001). One‐way ANOVA followed by Dunnet's post hoc (D, E, G, H), between groups of the same genotype. Asterisks represent differences between treatments and vehicle groups in the same genotype (**p < 0.01; ***p < 0.001; ****p < 0.0001). NG2, neural/glial antigen 2; MBP, myelin basic protein; T3‐WT, Wild‐type (WT) OPC cultures treated with triiodothyronine (T3).

Using the NSC‐derived OPC model we analyzed the differentiation/maturation of OPCs at 6 DIVs after T3‐mediated induction of differentiation (Figures 3A–C and S2). We confirmed a strong impairment of OPC differentiation in the *Rxrg* ^*−/−*^ cultures, as proven by a high percentage of NG2‐positive precursors (Student's *t*‐test, *p* < 0.0001; Figure 3A) and a low number of mature oligodendrocytes identified by MBP (Student's *t*‐test, *p* < 0.0001; Figure 3B). Representative images of NG2‐ and MBP‐labeled WT and *Rxrg* ^*−/−*^ cultures are shown in Figure 3C. These data further support the conclusion of a block of *Rxrg* ^*−/−*^ OPC differentiation revealed in our previous study, in which OPC differentiation was monitored using a combination of distinct protein markers: APC, Olig1, Olig2 and MBP (Baldassarro et al. 2019).

Due to the clear upregulation of the SHH pathway in *Rxrg* ^*−/−*^ OPCs, both at basal level and after 24 h of T3 exposure, we hypothesize a key role of this pathway in the events leading to impaired differentiation/maturation. Indeed, SHH signaling through both genomic and non‐genomic pathways plays an important role in OPC differentiation. Importantly, hyperactivity of SHH signaling was reported to impede NSC‐derived oligodendrogenesis (see Introduction). To test the hypothesis of a central role of SHH upregulation in *Rxrg* ^*−/−*^ OPC differentiation block, we pharmacologically downregulated this pathway using two different inhibitors, cyclopamine or GANT61, chosen because they act at different levels of the SHH pathway: (i) cyclopamine inhibits Smoothned (Smo), an immediate effector of SHH‐Patched1 activation; (ii) GANT61 acts as an inhibitor of GLI1, a transcriptional effector of SHH‐Patched1‐Smo activation (Figure S2). We treated both WT and *Rxrg* ^*−/−*^ NSC cultures with GANT61 or cyclopamine at 10 μM, simultaneously to T3 exposure (DIV 0), for 3 days, and analyzed OPC differentiation and maturation at 6 DIVs.

Inhibition of the SHH pathway significantly affected the percentage of NG2‐positive OPCs in both WT (One‐Way ANOVA, *F*(2.42) = 18.59, *p* < 0.0001) and *Rxrg* ^*−/−*^ (One‐Way ANOVA, *F*(2.42) = 70.29, *p* < 0.0001) cultures (Figure 3D): in WT and *Rxrg* ^*−/−*^ both treatments led to a reduction in the percentage of OL precursor cells (Dunnett's post‐test; WT, GANT61, *p* < 0.0001, cyclopamine, *p* = 0.0031; *Rxrg* ^*−/−*^, GANT61, *p* < 0.0001, cyclopamine, *p* < 0.0001). However, whereas in WT cultures GANT61 treatment resulted in a decrease of the percentage of mature MBP‐positive OLs (One‐Way ANOVA, *F*(2.42) = 30.29, *p* = 0.0001; Dunnet's post‐test, GANT61, *p* < 0.0001), both molecules normalized their number in *Rxrg* ^*−/*^cultures (One‐Way ANOVA, *F*(2.42) = 66.42, *p* < 0.0001; Dunnett's post‐test, GANT61, *p* < 0.0001; cyclopamine, *p* < 0.0001) (Figure 3E). Representative pictures of the cultures are included in Figure 3F.

Thus, in addition to compromised differentiation of *Rxrg* ^*−/−*^ OPCs, we also observed an impairment in the maturation of the *Rxrg* ^*−/−*^ NSC‐derived oligodendrocytes which was not studied previously. IMARIS‐aided voxel‐based analysis of the MBP+ mature OLs at 6 days after the induction of differentiation allowed to build an isosurface for each cell to measure the oligodendrocyte spider web‐shaped net as a correlate of cell volume (Figures 3G–I and S3). We used this image tool to further investigate whether inhibition of the SHH pathway restores the maturation process of mutant OLs. Both treatments increased the volume of the isosurface structures in *Rxrg* ^*−/−*^ (One‐Way ANOVA, *F*(2.42) = 24.51, *p* < 0.0001; Dunnett's post‐test; GANT61, *p* < 0.0001; cyclopamine, *p* < 0.0001), indicating improved and normalized myelination, as it was comparable to myelination of vehicle‐treated WT OLs (Figures 3G and S3). Surprisingly, WT OPCs displayed a reduction of the isosurface of mature OLs after GANT61 or cyclopamine treatment (One‐Way ANOVA, *F*(2.42) = 11.03, *p* = 0.0001; Dunnett's post‐test, GANT61, *p* = 0.0015; cyclopamine, *p* = 0.0001), possibly indicating an impaired myelination process (Figure 3G). The same results were obtained by Sholl analysis, which revealed reduction of the intersections in WT OLs (One‐Way ANOVA, *F*(2.42) = 12.90, *p* < 0.0001; Dunnett's post‐test, GANT61, *p* < 0.0001; cyclopamine, *p* < 0.0001) (Figure 3H). Representative pictures of IMARIS software elaborations are included in Figure 3I.

Additional, dose–response analyses of the two inhibitors (2.5, 5, and 10 μM) indicated that lower doses of cyclopamine or GANT61 are efficient in increasing OPC differentiation and myelination, although 10 μM allowed complete normalization of these processes in *Rxrg* ^*−/−*^ OPCs (Figure S4).

Since T3 is the signal switching OPCs from a proliferative state to a differentiation commitment, and as we previously demonstrated that *Rxrg* ^*−/−*^ OPCs fail to exit the cell cycle in response to T3 exposure (Baldassarro et al. 2019), we investigated if inhibition of the SHH pathway may also normalize cell cycle exit of *Rxrg* ^*−/−*^ OPCs in response to T3. To do so, we quantified the subpopulation of PDGFRa+ OPCs, which display mitotic activity identified by the expression of Ki67. We found that inhibition of the SHH pathway by either antagonist restores the ability of *Rxrg* ^*−/−*^ OPCs to exit the cell cycle, thereby allowing their differentiation (Figure S5).

### 3.5 Hyperactivation of the SHH Pathway in Sufficient to Induce a Differentiation Block in WT NSC‐Derived OPCs

**FIGURE 4:**
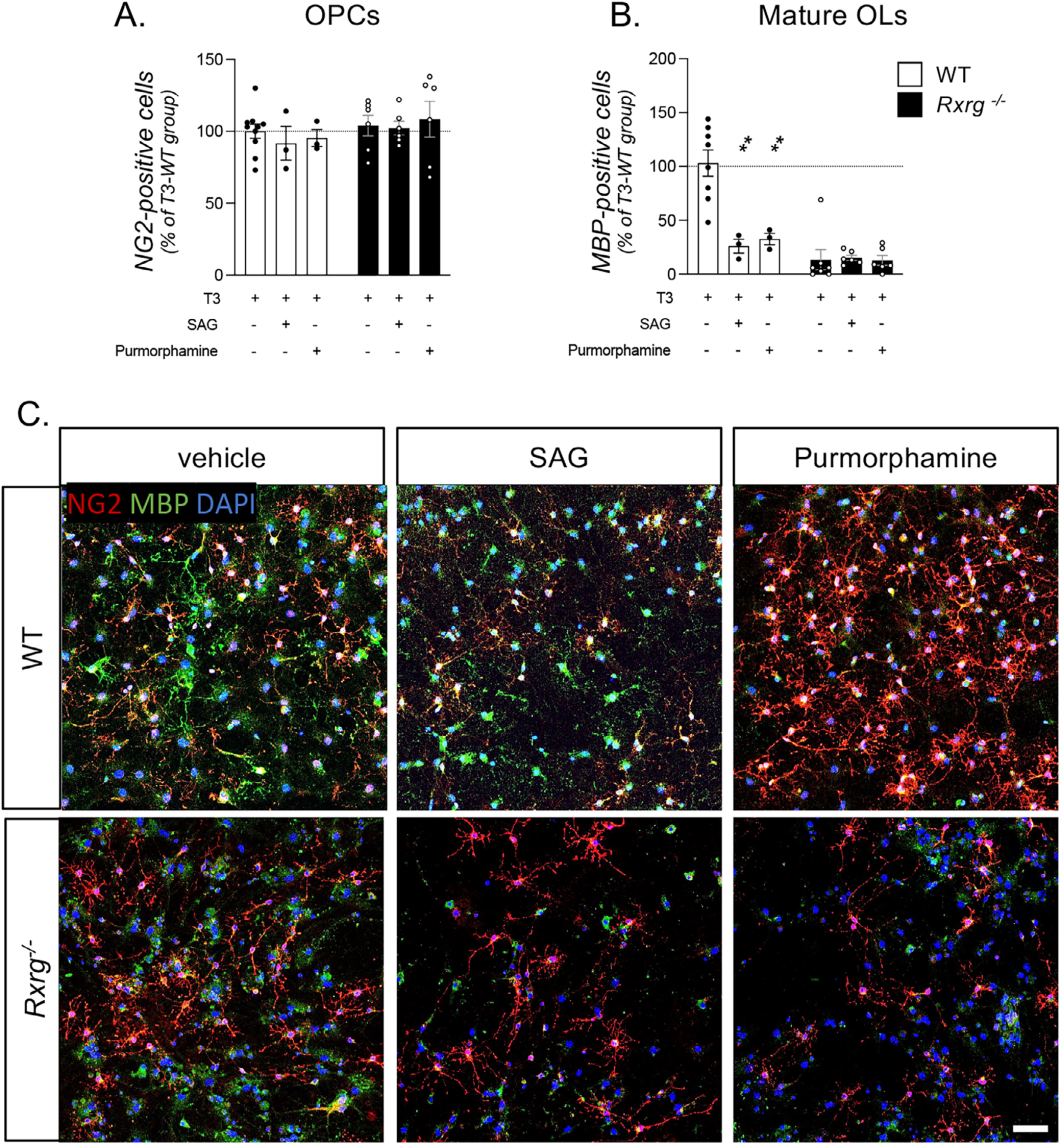
OPC differentiation is impaired in WT cultures when the SHH pathway is overactivated. (A, B) Graphs show the percentage of OPCs (A, NG2‐positive cells) and mature oligodendrocytes (B, MBP‐positive cells) in WT and *Rxrg* ^*−/−*^ cultures at 6 DIVs after T3 exposure, treated for 3 DIVs with vehicle, SAG or purmorphamine. Data are shown as percentage of the WT group treated with vehicle (horizontal dotted line, 100%). (C) Representative picture of OPCs and mature oligodendrocytes in WT and Rxrg^−/−^ cultures treated with vehicle, SAG or purmorphamine. Scale bar: 25 μm. Statistical analyses: One‐way ANOVA followed by Dunnet's post hoc (D, E, G, H), between groups of the same genotype. Asterisks represent differences between treatments and vehicle groups in the same genotype (**p < 0.01). NG2, neural/glial antigen 2; MBP, myelin basic protein; T3‐WT, Wild‐type (WT) OPC cultures treated with triiodothyronine (T3).

Whereas restoration of the differentiation and myelination potential of *Rxrg* ^*−/−*^ OPCs by pharmacological inhibition of the SHH pathway supports the idea of a critical, “necessary” contribution of hyperactive SHH signaling to the differentiation block associated with loss of *Rxrg* function, we asked whether hyperactive SHH signaling can be a “sufficient” condition to induce such a block of differentiation in the case of WT OPCs. To this end, we used two SHH agonists, SAG or purmorphamine (Figure 4A–C), to overactivate SHH signaling in both WT and *Rxrg* ^*−/−*^ NSC‐derived OPCs (Figures S6 and S7). Treatment with either agonist did not modify the percentage of NG2‐expressing OPC cells in both genotypes (Figure 4A). However, WT cultures exposed to SAG or purmorphamine displayed a clear deficit in differentiating (MBP‐positive) cells (One‐Way ANOVA, *F*(2.11) = 11.77, *p* = 0.0018; Dunnett's post‐test; GANT61, *p* = 0.0038; cyclopamine, *p* = 0.0069), while no further decrease of differentiation was observed in *Rxrg* ^*−/−*^ cells, most probably due to floor effect (Figure 4B).

### 3.6 Compromised Differentiation of Post‐Natal Primary Rxrg^−/−^ OPCs Is Associated With Dysregulated SHH Signaling

**FIGURE 5:**
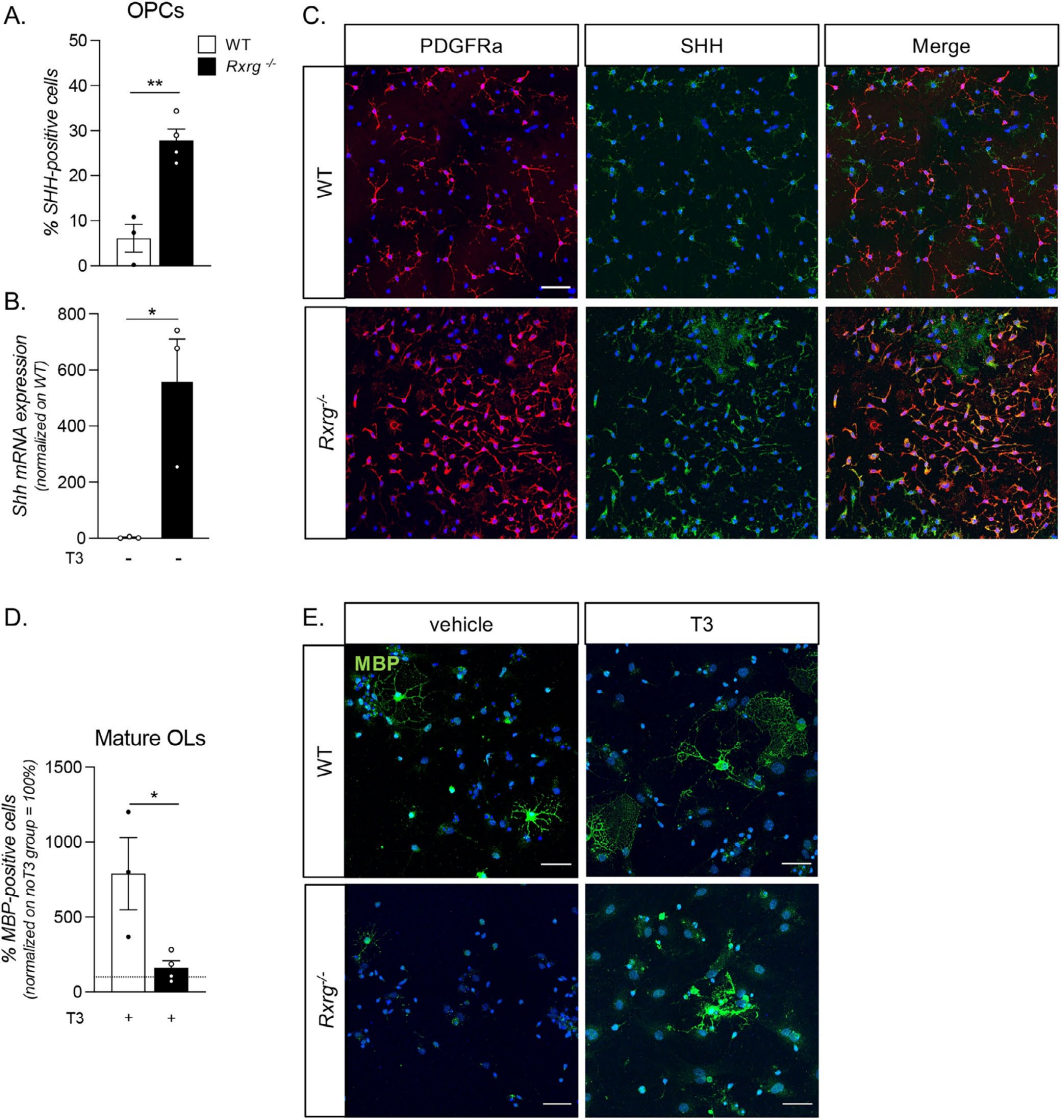
SHH signaling is dysregulated and differentiation is impaired in post‐natal *Rxrg* ^*−/−*^ OPC cultures. (A) Graph shows *Shh* gene expression in WT and *Rxrg* ^*−/−*^ primary OPC cultures. (B) Graph shows the percentage of SHH‐positive OPCs in WT and *Rxrg* ^*−/−*^ post‐natal primary OPC cultures. (C) Representative picture of WT and *Rxrg* ^*−/−*^ primary OPC (PDGFRa‐positive cells) undifferentiated cultures (no T3) double stained for SHH expression. Scale bar: 25 μm. (D) Graph shows the percentage of mature OLs in WT and *Rxrg* ^*−/−*^ post‐natal primary OPC cultures after exposure for 3 DIVs to the differentiation trigger T3. (E) Representative picture of WT and *Rxrg* ^*−/−*^ primary differentiated mature OLs (MBP‐positive cells). Scale bar: 25 μm. MBP, myelin basic protein; PDGFRa, platelet‐derived growth factor receptor alpha; T3, triiodothyronine; SHH, sonic hedgehog.

To test whether RXRg‐dependent dysregulation of *Shh* expression and associated deficit of OPC differentiation are specific only to NSC‐derived OPCs of embryonic origin, or may also occur post‐natally, we analyzed the expression of SHH mRNA and protein in post‐natal OPCs isolated from P7 brains and cultured in vitro. In comparison to WT, the *Rxrg* ^*−/−*^ OPC cultures displayed significantly increased *Shh* expression at the mRNA level (Student's *t*‐test, *p* = 0.0219; Figure 5A), which was further reflected by increased numbers of SHH‐expressing cells by immunocytochemistry (Student's *t*‐test, *p* = 0.0027; Figure 5B,C).

Similarly to embryonic NSC‐derived OPCs, post‐natal primary OPC cultures were exposed to the physiologic differentiation stimulus (T3). Whereas a marked increase of MBP‐positive cells was observed in WT cultures treated with T3, such increase was only marginal in *Rxrg* ^*−/−*^ cells (Student's *t*‐test, *p* = 0.0299; Figure 5D). Representative pictures of MBP‐positive cells (Figure 5E) also illustrate that the few MBP‐positive cells in *Rxrg* ^*−/−*^ cultures do not present the classical spider‐net shape morphology typical of mature OLs.

## 4 Discussion

Thyroid hormone (T3) is the main driver of OPC differentiation, both in the case of developmental myelination and in the adult remyelination process (Fukushima et al. 2015; Michalski and Kothary 2015; Yeung et al. 2014). The complex machinery underlying its final effect, that is, the production of a mature and functional oligodendrocyte, is yet poorly understood and is presumably regulated by various molecular players.

Since Raff and collaborators elucidated the role of T3 in driving OPCs out from the cell cycle (Barres et al. 1994) after a defined number of replication rounds (Billon et al. 2001, 2002), the effects of this hormone and its NRs on gene regulation have been deeply investigated (Baxi et al. 2014; Casaccia‐Bonnefil and Liu 2003; Grøntved et al. 2015). It turned out that T3 is directly involved in differentiation induction (Tokumoto et al. 2002), regulating the expression of genes/proteins involved in cell cycle control (Puzianowska‐Kuznicka et al. 2006).

T3 action on gene regulation has been described for several TH‐responding cell types, among various tissues in mammalian organisms. Research has aimed to find gene targets of TRs and their possible heterodimerization partners (Dong et al. 2009), pointing to RXRg as the main NR participating in the OPC differentiation process (Baldassarro et al. 2019). In line with this possibility, RXRg emerged as a key positive regulator of remyelination (Huang et al. 2011). Indeed, almost complete loss of RXRg expression has been observed in OPCs localized within chronic lesions of the spinal cord (Huang et al. 2011), which was suggested to underlie the differentiation block in OPCs, a pathological mechanism suggested as the main culprit of the remyelination failure in multiple sclerosis (Skaper 2019).

In the present study, we investigated the mechanism through which *Rxrg* contributes to T3‐induced OPC differentiation using a well‐established and robust experimental model of NSC‐derived OPCs generated from fetal mouse brain (Baldassarro 2021; Baldassarro et al. 2017, 2020). This experimental model was preferred to primary and purified post‐natal OPCs to better mimic the developmental myelination process, with a focus on the oligodendroglial lineage specification as described (Baldassarro et al. 2019). Using bulk RNAseq analyses we observed that transcriptional changes at 24 h after T3 treatment are compromised by about 50% in *Rxrg* ^*−/−*^ as compared to WT NSC‐derived OPCs. This reduction reflects loss of T3‐mediated transcriptional control of TRs over a wide range of genes (approximately 50%) identified by Zekri et al. (2022) as direct transcriptional targets of TRa and/or TRb in mouse brain. This observation is in coherence with RXRg contributing to TR activities as an obligatory heterodimerization partner, suggesting that RXRg‐TR controlled transcriptional programs are key for OPC differentiation to OLs. Functional annotation of T3 DEGs revealed that several distinct programs assuring successful OPC differentiation to oligodendrocytes are compromised in *Rxrg* ^*−/−*^ OPCs. This includes, in particular, T3‐dependent suppression of neuronal differentiation (e.g., downregulation of *Dlx1, Dlx2, Tal1, Slit1, GDF10*) and negative regulators of oligodendrocyte differentiation and myelination (e.g., downregulation of *Slit1*) (Achim et al. 2013; Deboux et al. 2020; Li et al. 2015; Petryniak et al. 2007). In parallel, significant induction of *Zif536*, a transcription factor necessary and sufficient to induce oligodendrocyte fate in vitro and in vivo (Dugas et al. 2006; Yang et al. 2013), was observed only in T3‐treated WT, but not *Rxrg* ^*−/−*^ cells. In contrast, among genes upregulated in T3‐treated *Rxrg* ^*−/−*^ (but not WT) OPCs were genes related to *axon* and *neuron projection development* (Se*ma4d, Plxnd1, Ephb1, Sema3d, Tmem108, Gap43, Cntn2, Cck, Pou4f1, Mag, Grm7, Ugt8a, Mbp*), coherent with a shift of these cells toward a neuronal cell fate. Some of these genes were identified as direct TR transcriptional targets (*Sema4d, Plxnd1, Cntn2, Mbp*). However, absence of *Rxrg* did not completely abolish the oligodendrogenic program as several genes relevant to OL cell fate (e.g., *Opalin, Nkx6.2, Mbp*, or *Mag*) were induced by T3 both in WT and *Rxrg* ^*−/−*^ OPCs. However, such changes were not sufficient to assure successful OPC differentiation and myelination, as indicated by increased number of NG2+ or PDGFRa+ OPCs at the expense of MBP+ differentiated Ols in *Rxrg*‐deficient cell cultures (present work and Baldassarro et al. 2019). Overall, such data indicate that a default action of T3‐mediated TR activities could be driving some of the initial steps of cell differentiation toward a neuronal/oligodendrocyte cell fate, whereas RXRg interaction with TRs would aim at refining this cell fate toward the oligodendrocyte lineage.

Why an OL fate was not attained in *Rxrg* ^*−/−*^ cells despite partial activation of the oligodendrogenic program may also have multiple origins. Some of them were revealed by analyzing DEGs in *Rxrg* ^*−/−*^ vs. WT OPCs prior to T3 treatment. We observed, for instance, that *Opalin, Nkx6.2, Mbp, Mog, Mobp*, or *Mag*, all genes required for oligodendrogenesis, were strongly downregulated in *Rxrg*‐null OPCs in the absence of T3. Thus, and even if T3 treatment would eventually stimulate their expression, it still remained significantly lower than in WT OPCs during the differentiation process. In addition, several genes involved in oligodendrogenesis were reduced in *Rxrg* ^*−/−*^ OPCs and remained insensitive to T3 treatment. These include, for example, *Plp1, Myrf, Lpar1, Dusp10, Cnp, Tppp, Mal, Tspan2, Tmem98, Dusp15*, and *Sirt2*. In parallel to compromised oligodendrogenesis, several biological processes were significantly upregulated in *Rxrg* ^*−/−*^ OPCs, pointing to cell motility, cardiovascular system development, extracellular matrix structure, and signaling receptor binding as abnormally upregulated gene ontology processes. A significant 6‐ to 11‐fold upregulation of *Shh* was associated with most of these GO terms.

We used a pharmacological approach to inhibit the SHH pathway at the level of Smo or Gli, using respectively cyclopamine or GANT61 treatment along with T3 treatment, in our experimental model. We found that both inhibitors were extremely efficient in normalizing differentiation of *Rxrg* ^*−/−*^ OPCs, but also potentially for myelination of newly generated *Rxrg* ^*−/−*^ OLs. Such data indicate that the hyperactive SHH signaling pathway is a causal, rather than coincidental mechanistic factor in mediating the differentiation and myelination block in the absence of *Rxrg*. We also demonstrated that hyperactivity of the SHH pathway is sufficient to inhibit differentiation and subsequent myelination potential of WT OPCs and OLs. Indeed, activation of SHH signaling in WT OPCs using either of two distinct agonists, purmorphamine or SAG, along with T3 treatment, each led to increased numbers of OPCs and a significant decrease of MBP+ OLs. Whereas hyperactive SHH signaling is clearly inhibitory for oligodendrogenesis of WT and *Rxrg* ^*−/−*^ OPCs, as demonstrated in previous work (Nocera et al. 2024), SHH hypoactivity also appeared detrimental for oligodendrogenesis as documented by a strong increase of undifferentiated NG2+ OPCs at the expense of differentiating MBP+ OLs. Globally, our data indicate that oligodendrogenesis is highly sensitive to the dosage of SHH signaling, with an optimal level of such signaling required for efficient differentiation and myelination processes. Importantly, RXRg emerges as a new regulator of optimal SHH signaling in OPCs. These data might be particularly relevant for oligodendrocytes generated from NSCs, as previous studies reported similar inhibitory effects of SHH pathway activation in neuroblasts or NSC‐derived OPCs (Namchaiw et al. 2019; Radecki et al. 2020; Samanta et al. 2015). However, it remains uncertain whether RXRg control of SHH is also relevant to the pool of quiescent OPCs, as activation of SHH signaling mostly had pro‐differentiating effects in these cells (Emamnejad et al. 2022; Loulier et al. 2006).

How RXRg controls *Shh* expression is not clear, but it is difficult to exclude that it acts as a transcriptional repressor of *Shh* expression or that it only mediates or preconditions response to T3 activities. Indeed, whereas nothing is known about direct transcriptional control of *Shh* by RXRs, a TR‐mediated transcriptional control of *Shh* by T3 was proposed to modulate *Shh* levels or levels of its upstream mediators *Ptch* and *Smo* (Bernal 2017; Desouza et al. 2011). Moreover, *Shh* expression is reduced in the cerebellum of hypothyroid rats (Hasebe et al. 2008) and, in neuronal cultures, T3 exposure leads to a rapid histone acetylation at the level of the *Shh* gene promoter (Desouza et al. 2011). Furthermore, a non‐transcriptional, T3‐dependent pathway mediated by integrins has also been suggested to control *Shh* expression (Lee and Petratos 2016). Finally, the overexpression of ECM‐related genes and integrins that we observed in *Rxrg* ^*−/−*^ OPCs may reflect hyperactivity of Shh signaling as previously suggested in the context of neural tube morphogenesis (Fournier‐Thibault et al. 2009), but may also synergize and/or modulate SHH signaling to control OPC differentiation (Jägers and Roelink 2021; Pons and Martí 2000; Urbanski et al. 2016; Wheeler and Fuss 2016), and potentially enhance OPC differentiation block in demyelinating conditions (Bighinati et al. 2021; Ghorbani and Wee Yong 2021; de Jong et al. 2020). Clearly, it will be of interest to further investigate the impact of *Rxrg* ^*−/−*^‐linked ECM alterations.

Previous developmental studies reported that SHH activity is strictly regulated during myelination, including through the regulation of downstream mediators such as Lrp‐2 (*megalin*). Specifically, Lrp‐2 is highly expressed during OPC proliferation and migration phases, while being gradually downregulated during differentiation (Ortega et al. 2012). In agreement with deficient differentiation and persistent proliferation of *Rxrg* ^*−/−*^ OPCs, Lrp‐2 was found to be overexpressed in *Rxrg* ^*−/−*^ samples. This effector might thus be involved in the observed differentiation block.

Finally, since many OPCs generated during the early waves of developmental oligodendrogenesis are eliminated (Kessaris et al. 2006) and only some persist in postnatal life (Marques et al. 2018), it is debatable whether the effects of RXRg inactivation may be restricted to OPCs generated from embryonic NSCs in vitro and would not be seen in postnatal OPCs. Our data obtained from postnatal OPCs advocate against this possibility. MACS‐sorted OPCs from brains of one‐week‐old *Rxrg* ^*−/−*^ mice displayed deficient differentiation and maturation similar to that observed in embryonic NSC‐derived OPCs. Likewise, these cells also displayed increased SHH expression before or after T3 treatment, reflecting hyperactivity of its signaling in postnatal *Rxrg* ^*−/−*^ OPCs.

In conclusion, this study revealed that both in NSC‐derived and post‐natal OPCs, RXRg controls *Shh* expression to assure its optimal signaling required for successful differentiation and subsequent myelination potential of OPCs and OLs. We demonstrated that SHH pathway hyperactivity is sufficient in WT OPCs to induce a differentiation block, whereas in *Rxrg* ^*−/−*^ OPCs it is a causal, necessary factor impeding their differentiation and myelination. Our data point to the necessity of a tight regulation of SHH signaling to assure differentiation of NSC‐derived OPCs, as both hyper‐ and hypo‐activity of this pathway were incompatible with successful oligodendrogenesis of WT OPCs. Relevance of the TH‐RXR‐SHH regulatory axis may span beyond control of oligodendrogenesis: in particular, it might be relevant for carcinogenesis and would place compromised RXR signaling as a central, instructive signal for cancer progression. As an example, downregulation of RXRg expression is associated with poor prognosis in thyroid carcinoma (Song et al. 2025) and this effect could more widely reflect RXR involvement in regulation of cellular differentiation and programmed cell death in various types of cancers, possibly via RXR heterodimers with TRs, RARs (Kim and Cheng 2013; Schiera et al. 2021; Simon et al. 2002) or PPARs (Shankaranarayanan et al. 2009). Our present data support the idea of a requirement of RXRg for maintenance of signaling through TR, an essential tumor suppressor gene (Kim and Cheng 2013), and complement this image by suggesting that enhanced SHH expression and signaling known to promote cancer stem cell features and development and/or progression of multiple types of cancer (Reslinger and Plateroti 2025; Takebe et al. 2011; Williamson et al. 2016) may also result from compromised RXR signaling. Such data encourage future investigations to functionally dissect the role of the TR‐RXR‐SHH axis in cancer.

## Author Contributions

Conceptualization: V.A.B. and W.K. Data curation: V.A.B., Q.B., and W.K. Formal analysis: V.A.B. and W.K. Funding acquisition: V.A.B., L.C., and W.K. Investigation: V.A.B., Q.B., and V.F. Methodology: V.A.B., Q.B., and W.K. Project administration: L.C. and W.K. Resources: W.K. Supervision: V.A.B. L.C., and W.K. Validation: V.A.B. and W.K. Visualization: V.A.B. and W.K. Writing – original draft: V.A.B. and W.K. Writing – review and editing: V.A.B., Q.B., L.C., and W.K.

## Funding

This work was supported by Fondation pour l'Aide à la Recherche sur la Sclérose en Plaques; project: Role of RXRg in T3‐mediated oligodendrocyte differentiation.

## Conflicts of Interest

The authors declare no conflicts of interest.

## Supporting information


**Figure S1:** Heat map representation of transcriptional changes determined by RNAseq in cultured WT and Rxrg^−/−^ OPCs at 24 h after vehicle and T3 treatment. T3, triiodothyronine; WT, Wilde type.
**Figure S2:** Timeline and strategy of SHH pathway treatments. (A) NSCs‐derived OPCs culture protocol timeline and growth factors. (B) Treatment of SHH pathway inhibitors. (C) Treatment of SHH pathway activators. (D) Schematic representation of SHH activators and inhibitors targets. bFGF, basic fibroblst growth factor; DIV, day in vitro; DMSO, dimethyl sulfoxide; EGF, epidermal growth factor; Gli1, glioma‐associated oncogene; OL, oligodendrocyte; OPC, oligodendrocyte precursor cell; PDGF, platelet derived growth factor; Ptch1, patched 1; SAG, smoothened agonist; Shh, sonic hedgehog; Smo, smoothned; T3, triiodothyronine; WT, Wilde type.
**Figure S3:** Analysis of Wt and Rxrg^−/−^ oligodendrocyte maturation. (A) Oligodendrocytes were analyzed after 6 DIVs of T3 exposure. Cultures were stained for MBP expression and confocal images were acquired and processed by the voxel‐based image analysis using the IMARIS software. Using the MBP‐based fluorescence the software is able to create an isosurface isolating the volume of each single mature oligodendrocyte. The isosurface was then used as a mask to build the network (filament algorithm) of the cell body to analyze the complexity of the spider web‐shaped net. (B, C) Graphs show the quantification of the cell body volume (B) and the Sholl analysis (C) of each analyzed cell. (D–G) Representative images of MBP stained (D, F) and IMARIS elaborated (E, G) images of Wt (D, E) and Rxrg^−/−^ (F, G) cells. (H–O) Representative images of Wt (H, I, L, M) and Rxrg^−/−^ (J, K, N, O) cells, treated with GANT61 (H—K) and cyclopamine (L–O), stained for MBP (H, J, L, N) and elaborated with IMARIS software (I, K, M, O) for the analysis of the cell body complexity (see main text Figure 4G–I). MBP, myelin basic protein; OL, oligodendrocyte; WT, wild type.
**Figure S4:** Dose–response analysis of SHH inhibitors GANT61 and cyclopamine. (A, B) Graphs show the quantification of NG2‐positive OPCs (A) and MBP‐positive mature oligodendrocytes (B) treated with T3‐only (black column) or together with GANT61/cyclopamine at different concentrations (gray columns). A control group cultured without T3 and SHH antagonist is also included (white column). (C) Representative images. Statistical analysis. One Way Anova followed by Dunnett's *t*‐test. Asterisks represent statistical significant differences in comparison with the T3‐only treated group (black column) (**p < 0.01; ****p < 0.0001). NG2, neuron‐glial antigen2; MBP, myelin basic protein.
**Figure S5:** Analysis of the replicating cells in Rxrg^−/−^ NSC‐derived OPC. (A) Graph represents the percentage of replicating OPCs (PDGFRa‐Ki67‐double positive cells) on the total number of OPCs (PDGFRa‐positive cells), in Rxrg^−/−^ cultures exposed or not to T3 and treated with vehicle, GANT61 or cyclopamine. (B) Representiative pictures of OPCs (PDGFRa positive cells), replicating cells (Ki67‐positive cells), and merged pictures representing the replicating OPCs (PDGFRa/Ki67‐double positive cells) in Rxrg^−/−^ cultures. Statistical analysis. One Way Anova followed by Dunnett's *t*‐test. Asterisks represent statistically significant differences in comparison with the T3‐only treated group (****p < 0.0001). Ki67, antigen Kiel 67; OPC, oligodendrocyte precursor cell; PDGFRa, platelet derived growth factor receptor alpha; T3, triiodothyronine.
**Figure S6:** Dose–response analysis of SHH agonist SAG. (A, B) Graphs show the quantification of NG2‐positive OPCs (A) and MBP‐positive mature oligodendrocytes (B) treated with T3‐only (black column) or together with SAG at different concentrations (gray columns) in Wt and Rxrg^−/−^ cultures. (C) Representative images. Scale bar = 20 μm. Statistical analysis. One‐Way Anova followed by Dunnett's *t*‐test. Asterisks represent statistically significant differences in comparison with the T3‐only treated group (black column) within the same genotype (***p < 0.001).MBP, myelin basic protein; NG2, neuron‐glial antigen2; OL, oligodendrocyte; OPC, oligodendrocyte precursor cell; SAG, smoothened agonist; T3, triiodothyronine; WT, wild type.
**Figure S7:** Dose–response analysis of SHH agonist purmorphamine. (A, B) Graphs show the quantification of NG2‐positive OPCs (A) and MBP‐positive mature oligodendrocytes (B) treated with T3‐only (black column) or together with purmorphamine at different concentrations (gray columns) in Wt and Rxrg^−/−^ cultures. (C) Representative images. Scale bar = 20 μm. Statistical analysis. One‐Way Anova followed by Dunnett's *t*‐test. Asterisks represent statistically significant differences in comparison with the T3‐only treated group (black column) within the same genotype (***p < 0.001). MBP, myelin basic protein; NG2, neuron‐glial antigen2; OL, oligodendrocyte; OPC, oligodendrocyte precursor cell; T3, triiodothyronine; WT, wild type.

**Table S1:** Table.

**Table S2:** Table.

## Data Availability

All the raw data are available in the public institutional research repository AMSActa, doi: https://doi.org/10.6092/unibo/amsacta/8345.
