# Supplementary material for: RXR Gamma Enables Oligodendrocyte Differentiation by Suppressing Sonic Hedgehog Signaling
Source: Glia. 2026 Apr 3;74(6):e70151. doi: 10.1002/glia.70151 (PMC13049363; doi:10.1002/glia.70151)
Supplement: Supplementary file 1 — Figure S1: Heat map representation of transcriptional changes determined by RNAseq in cultured WT and Rxrg−/− OPCs at 24 h after vehicle and T3 treatment. T3, triiodothyronine; WT, Wilde type. Figure S2: Timeline and strategy of SHH pathway treatments. (A) NSCs‐derived OPCs culture protocol timeline and growth factors. (B) Treatment of SHH pathway inhibitors. (C) Treatment of SHH pathway activators. (D) Schematic representation of SHH activators and inhibitors targets. bFGF, basic fibroblst growth factor; DIV, day in vitro; DMSO, dimethyl sulfoxide; EGF, epidermal growth factor; Gli1, glioma‐associated oncogene; OL, oligodendrocyte; OPC, oligodendrocyte precursor cell; PDGF, platelet derived growth factor; Ptch1, patched 1; SAG, smoothened agonist; Shh, sonic hedgehog; Smo, smoothned; T3, triiodothyronine; WT, Wilde type. Figure S3: Analysis of Wt and Rxrg−/− oligodendrocyte maturation. (A) Oligodendrocytes were analyzed after 6 DIVs of T3 exposure. Cultures were stained for MBP expression and confocal images were acquired and processed by the voxel‐based image analysis using the IMARIS software. Using the MBP‐based fluorescence the software is able to create an isosurface isolating the volume of each single mature oligodendrocyte. The isosurface was then used as a mask to build the network (filament algorithm) of the cell body to analyze the complexity of the spider web‐shaped net. (B, C) Graphs show the quantification of the cell body volume (B) and the Sholl analysis (C) of each analyzed cell. (D–G) Representative images of MBP stained (D, F) and IMARIS elaborated (E, G) images of Wt (D, E) and Rxrg−/− (F, G) cells. (H–O) Representative images of Wt (H, I, L, M) and Rxrg−/− (J, K, N, O) cells, treated with GANT61 (H—K) and cyclopamine (L–O), stained for MBP (H, J, L, N) and elaborated with IMARIS software (I, K, M, O) for the analysis of the cell body complexity (see main text Figure 4G–I). MBP, myelin basic protein; OL, oligodendrocyte; WT, wild type. Figure S4: [file GLIA-74-0-s003.pdf]

SUPPLEMENTARY MATERIALS: supplementary figures and results

RXR gamma enables oligodendrocytes differentiation by suppressing Sonic Hedgehog signaling.

Authors : Vito Antonio Baldassarro[1,2], Quentin Brassart[1], Valérie Fraulob[1], Laura Calzà[3], Wojciech Krezel[1]

[1]Institut de Génétique et de Biologie Moléculaire et Cellulaire, Centre National de la Recherche Scientifique UMR7104, Institut national de la santé et de la recherche médicale U 1258, Université de Strasbourg, 1 rue Laurent Fries, 67404, Illkirch-Graffenstaden, France

[2] Department of Veterinary Medical Science, University of Bologna, Ozzano dell'Emilia, Bologna, Italy

[3] Department of Pharmacy and Biotechnology, University of Bologna, Bologna, Italy

1. Rxrg-/- NSC-derived OPCs transcriptional changes

Vehicle and T3-treated Rxrg-/- OPCs show different transcriptional patterns (Supplementary Figure S1).

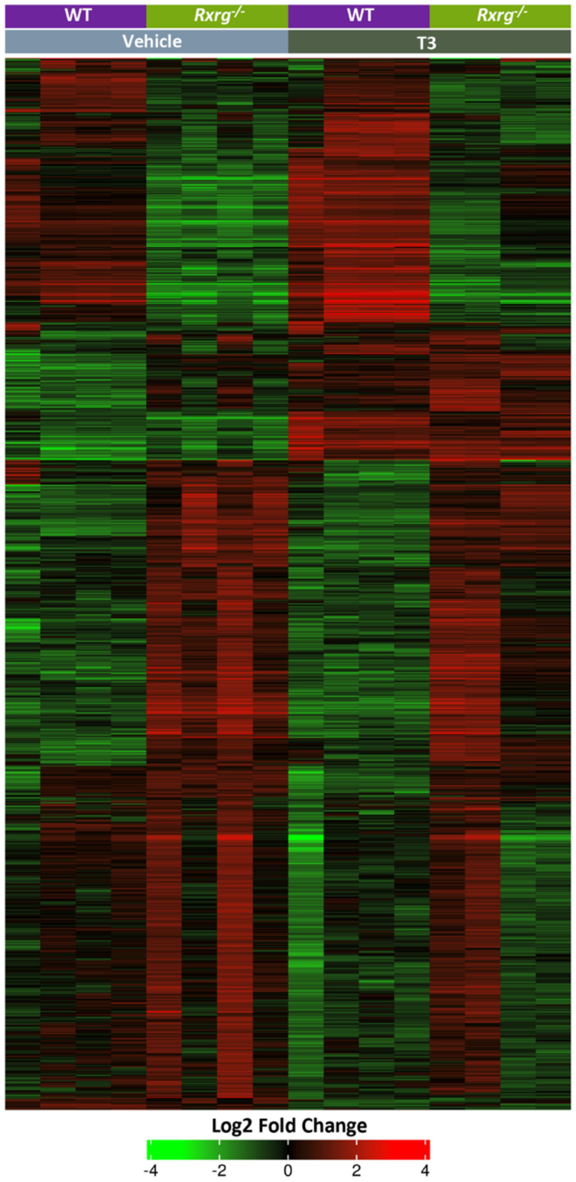

Figure Supplementary 1. Heat map representation of transcriptional changes determined by RNAseq in cultured WT and *Rxrg*$^{-/-}$ OPCs at 24 hours after vehicle and T3 treatment.
Abbreviations: T3, triiodothyronine; WT, Wilde type.

2. Protocol used to isolate/differentiate Rxrg-/- and WT NSC-derived OPCs and the molecular tools used to activate/inactivate the SHH pathway

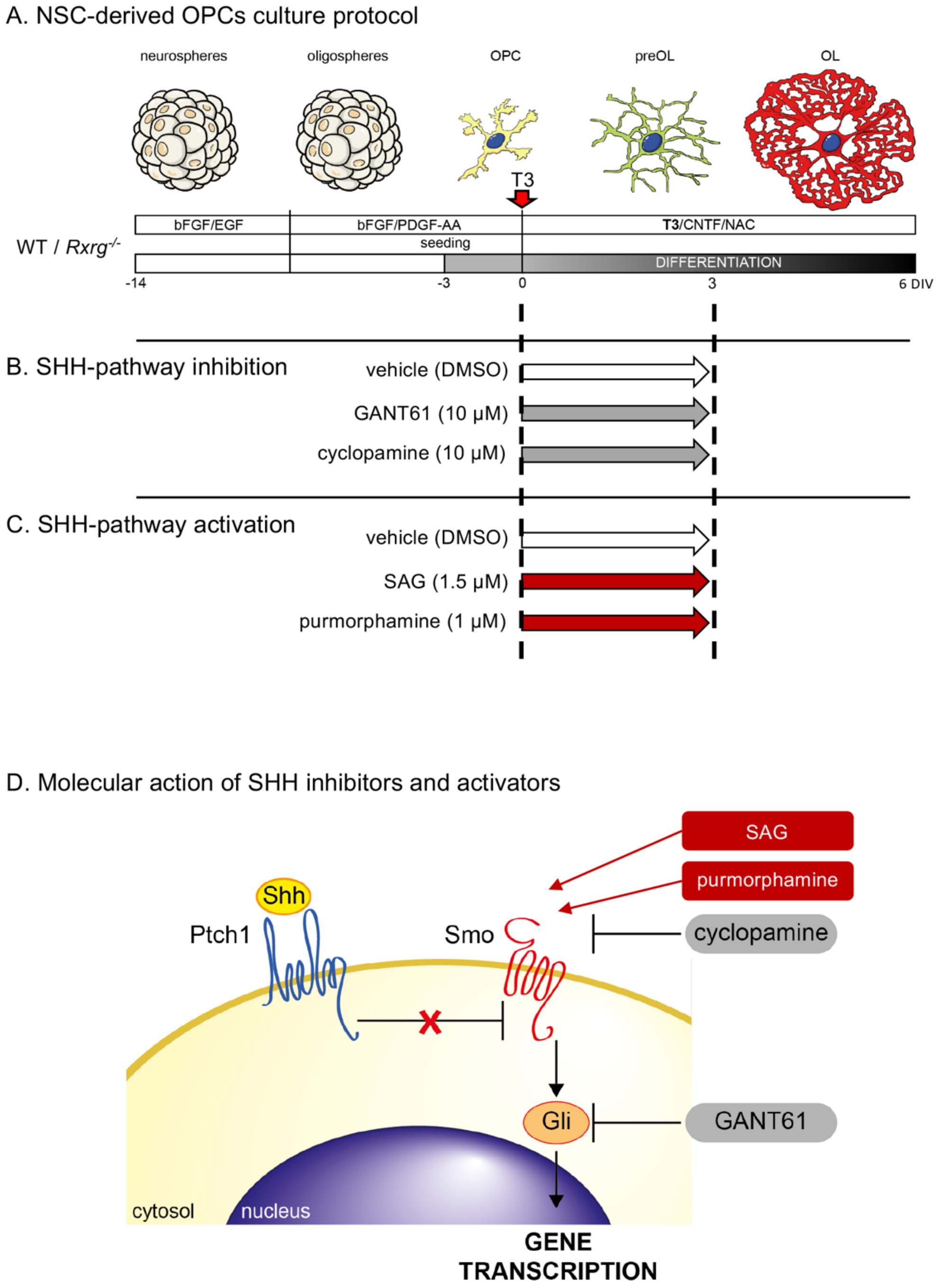

Figure Supplementary 2. Timeline and strategy of SHH pathway treatments. A) NSCs-derived OPCs culture protocol timeline and growth factors. **B)** Treatment of SHH pathway inhibitors. **C)** Treatment of SHH pathway activators. **D)** Schematic representation of SHH activators and inhibitors targets.

Abbreviations: bFGF, basic fibroblst growth factor; DIV, day in vitro; DMSO, dimethyl sulfoxide; EGF, epidermal growth factor; Gli1, glioma-associated oncogene; OL, oligodendrocyte; OPC, oligodendrocyte precursor cell; PDGF, platelet derived growth factor; Ptch1, patched 1; SAG, smoothened agonist; Shh, sonic hedgehog; Smo, smoothned; T3, triiodothyronine; WT, Wilde type.

3. Rxrg-/- NSC-derived OPCs show reduced maturation

The voxel-based analyses of Wt and *Rxrg*$^{-/-}$ OLs at 6 DIV of differentiation was performed by IMARIS software, using the isosurface built on the MBP fluorescence to isolate single cells and derive the structure of the spider-web shaped cell body (Supplementary Figure S2A). The analysis revealed that the few mature oligodendrocytes in *Rxrg-/-* cultures displayed approximately 4 fold reduction of cell body volume, indicating an impairment also in the maturation process(Student's t-test, $p < 0.0001$) (Supplementary Fig. S3B). Moreover, by tracking the filamentous components inside the MBP-positive extension of the oligodendrocyte cell body we measured the Sholl parameter which indicates the complexity of the net (Student's t-test, $p < 0.0001$) (Supplementary Fig. S3C). Representative images are included in the figure (Supplementary Fig. S3D-G). The reduction of the number of intersections in Sholl analysis indicate lower complexity of mature *Rxrg*$^{-/-}$ oligodendrocytes further supporting deficit of their maturation.

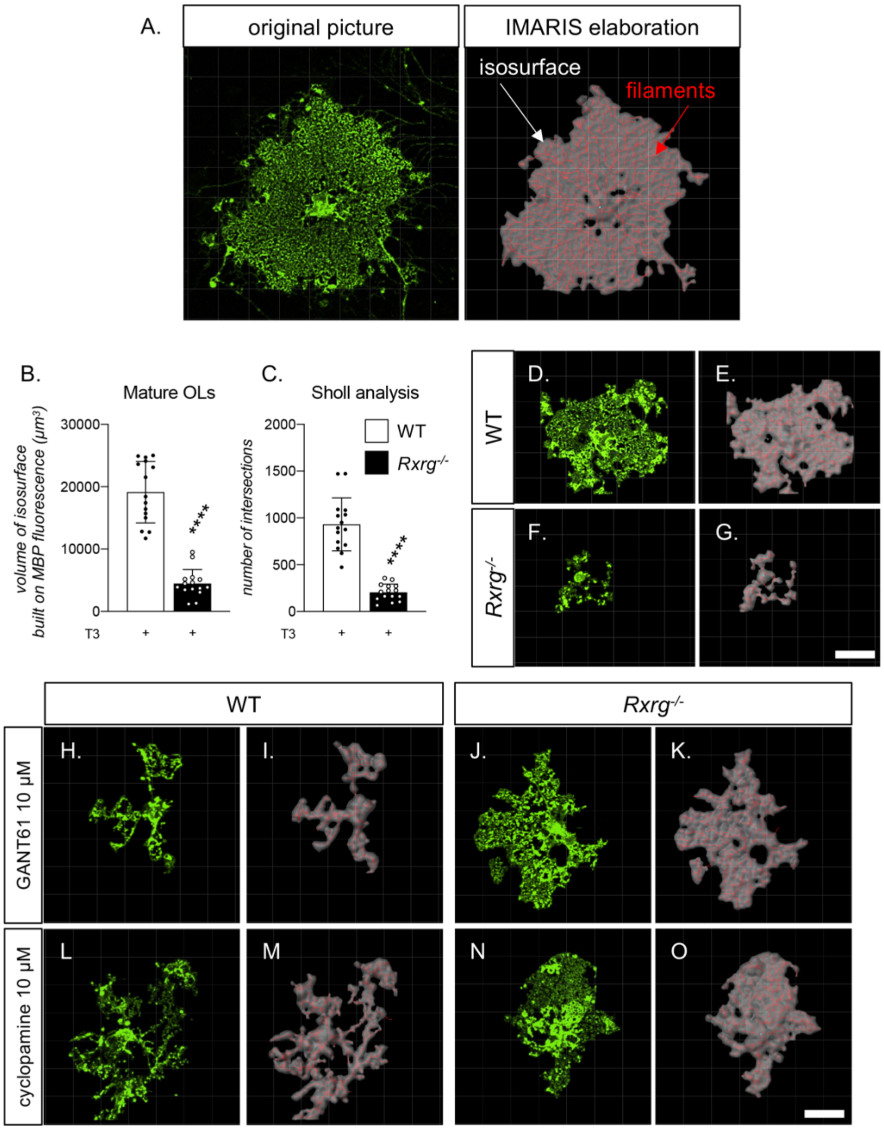

Figure Supplementary 3. Analysis of Wt and Rxrg-/- oligodendrocyte maturation.
(A) Oligodendrocytes were analyzed after 6 DIVs of T3 exposure. Cultures were stained for MBP expression and confocal images were acquired and processed by the voxel-based image analysis using the IMARIS software. Using the MBP-based fluorescence the software is able to create an isosurface isolating the volume of each single mature oligodendrocyte. The isosurface was then used as a mask to build the network (filament algorithm) of the cell body to analyze the complexity of the spider web-shaped net. (B – C) Graphs show the quantification of the cell body volume (B) and the Sholl analysis (C) of each analyzed cell. (D – G) Representative images of MBP stained (D, F) and IMARIS elaborated (E, G) images of Wt (D, E) and Rxrg-/- (F, G) cells. (H – O) Representative images of Wt (H, I, L, M) and Rxrg-/- (J, K, N, O) cells, treated with GANT61 (H – K) and cyclopamine (L – O), stained for MBP (H, J, L, N) and elaborated with IMARIS software (I, K, M, O) for the analysis of the cell body complexity (see main text Figure 4G-I).
Abbreviations: MBP, myelin basic protein; OL, oligodendrocyte; WT, wild type.

4. Dose-response curve of the SHH antagonists GANT61 and cyclopamine on Rxrg-/- NSC-derived OPC cultures

We tested both cyclopamine and GANT61 for three different doses (2.5, 5, 10 μM) following the standard protocol (Supplementary Figure S1). We compared all the results with the T3 exposed groups. For the NG2-positive OPCs, the cultures untreated with both T3 and SHH-antagonist resulted significantly different (One-Way ANOVA, $F(7,66) = 47.33$, $p < 0.0001$; Dunnett's post-test, $p < 0.0001$). Both GANT61 and cyclopamine treatments resulted in a significantly lower percentage of precursors only at 10 μM ($p < 0.0001$) (Supplementary Fig. S4A). For the quantification of mature MBP-positive OLs the treatment were also effective (One-Way ANOVA, $F(7,66) = 43.93$, $p < 0.0001$). For GANT61 5 and 10 μM generates an increase in differentiation ($p < 0.0001$), while the same effect was produced by all the three tested doses of cyclopamine (2.5 μM, $p = 0.0082$; 5 μM and 10 μM $p < 0.0001$) (Supplementary Fig. S4B). Representative images are included in Supplementary Figure S4C.

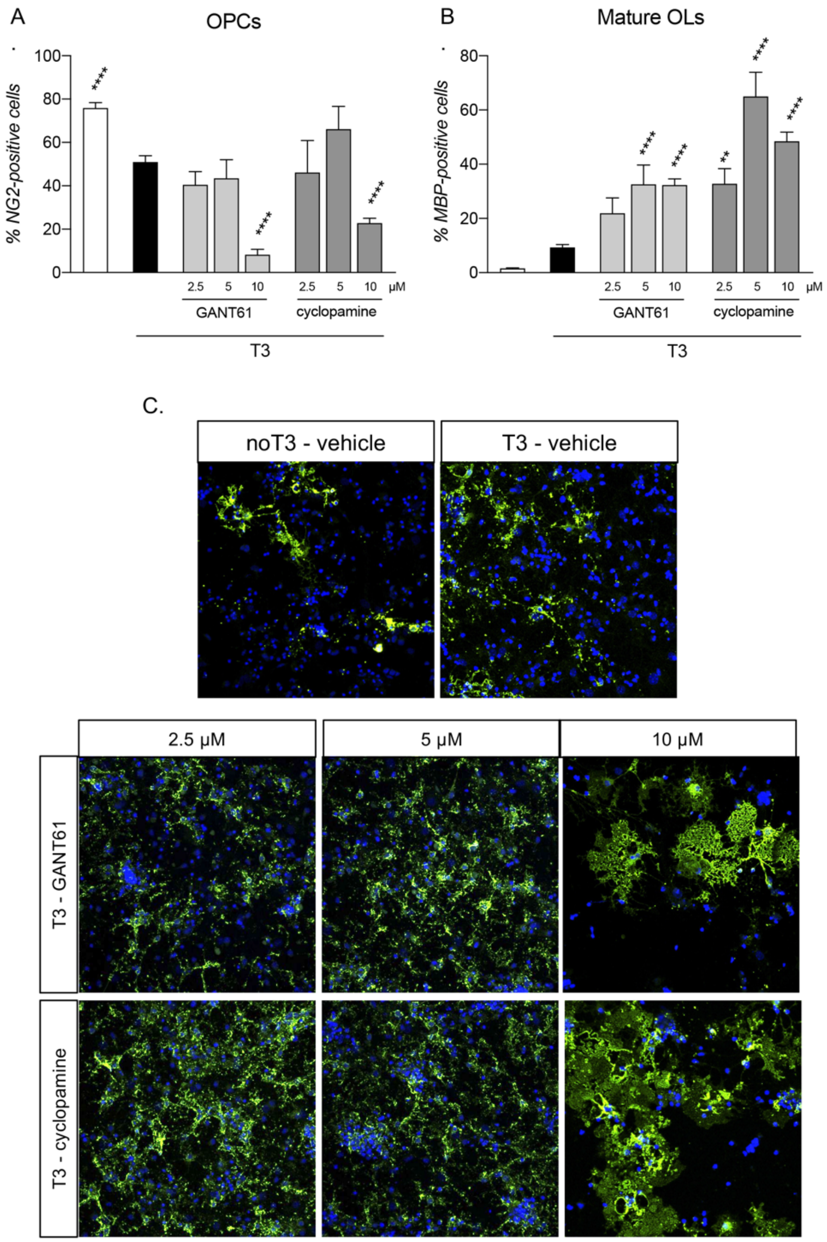

Figure Supplementary 4. Dose-response analysis of SHH inhibitors GANT61 and cyclopamine. (A - B) Graphs show the quantification of NG2-positive OPCs (A) and MBP-positive mature oligodendrocytes (B) treated with T3-only (black column) or together with GANT61/cyclopamine at different concentrations (gray columns). A control group cultured without T3 and SHH antagonist is also included (white column). (C) Representative images. Statistical analysis. One-Way Anova followed by Dunnett's t-test. Asterisks represent statistical significant differences in comparison with the T3-only treated group (black column) (** $p < 0.01$; **** $p < 0.0001$).
Abbreviations: NG2, neuron-glial antigen2; MBP, myelin basic protein.

5. Blocking the Shh pathway Rxrg-/- OPCs respond to T3-induced cell cycle exit

Since T3 is the signal switching the OPC from a proliferative state to a differentiation commitment, and we already demonstrated that Rxrg-/- fails to exit the cell cycle in response to T3 exposure (Baldassarro et al., 2019), we therefore investigated if the inhibition of Shh pathway normalizes also cell-cycle exit of Rxrg-/- OPCs in response to T3.

To do so, we used a double labeling with a OPC marker (PDGFRa) and a proliferation marker (Ki67) in Rxrg-/- cultures, and we than measured the percentage of proliferating OPCs (PDGFRa/Ki67 double positive) in the total number of OPCs (PDGFRa-positive cells). Both inhibitors treatment generated a restoring in the response capacity of Rxrg-/- OPCs to T3-mediated cell cycle exit (One-Way ANOVA, $F(3,46) = 60.12$, $p < 0.0001$; Dunnett's post-test, GANT61, $p < 0.0001$; cyclopamine, $p < 0.0001$) (Supplementary Fig. S5A). Representative pictures are included in Supplementary Figure 5B.

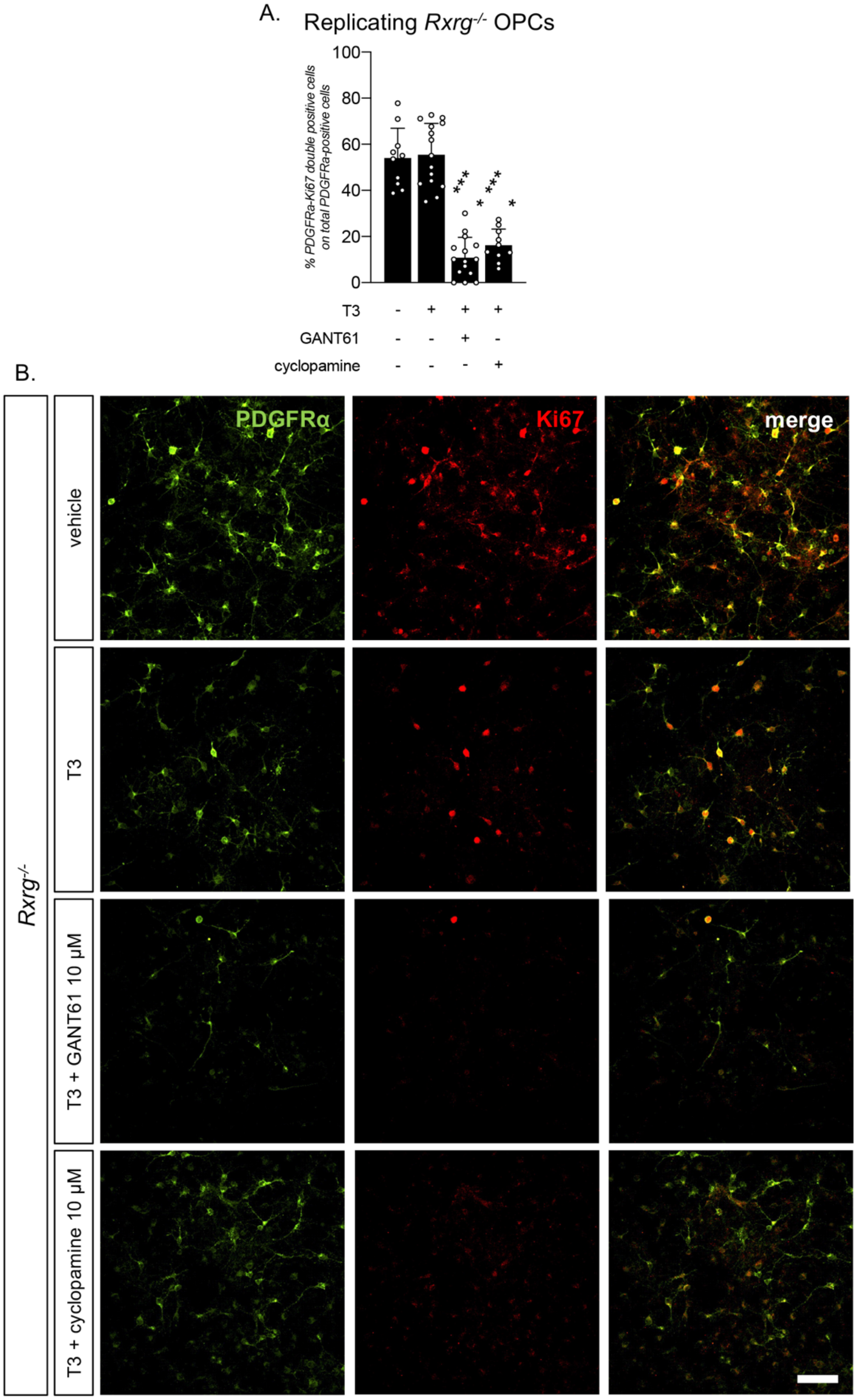

A.
Replicating Rxrg-/- OPCs
% PDGFRa-Ki67 double positive cells on total PDGFRa-positive cells
100
80
60
40
20
0
***
*
***
*
T3 - + + +
GANT61 - - + -
cyclopamine - - - +
B.
Rxrg-/-
vehicle
T3
T3 + GANT61 10 µM
T3 + cyclopamine 10 µM
PDGFRα
Ki67
merge

Figure Supplementary 5. Analysis of the replicating cells in Rxrg-/- NSC-derived OPC.

(A) Graph represents the percentage of replicating OPCs (PDGFRa-Ki67-double positive cells) on the total number of OPCs (PDGFRa-positive cells), in Rxrg-/- cultures exposed or not to T3 and treated with vehicle, GANT61 or cyclopamine. (B) Representiative pictures of OPCs (PDGFRa-positive cells), replicating cells (Ki67-positive cells), and merged pictures representing the replicating OPCs (PDGFRa/Ki67-double positive cells) in Rxrg-/- cultures. Statistical analysis. One-Way Anova followed by Dunnett's t-test. Asterisks represent statistically significant differences in comparison with the T3-only treated group (**** $p < 0.0001$).

Abbreviations: Ki67, antigen Kiel 67; OPC, oligodendrocyte precursor cell; PDGFRa, platelet derived growth factor receptor alpha; T3, triiodothyronine.

6. Dose-response curve of the SHH agonists SAG and purmorphamine on Wt and Rxrg-/- NSC-derived OPC cultures

We tested both SAG and purmorphamine for three different doses (SAG: 0.15, 0.5, 1.5 μM; purmorphamine: 0.1, 0.3, 1 μM) following the standard protocol (Supplementary Figure S2). We compared all the results with the T3 exposed groups.

For SAG treatments, no differences emerged from the analysis of the NG2-positive precursors in both genotypes (Supplementary Figure S6A). However, in WT cultures, MBP-positive cells showed a significant decrease in cultures treated with the two highest doses (One-Way ANOVA, $F(3,15) = 9.308$, $p = 0.0010$; Dunnet's post-test, 0.5 μM, $p = 0.0032$; 1 μM, $p = 0.0022$) (Supplementary Figure 6B). Representative images are included in Supplementary Figure S6C.

Also for purmorphamine treatments, no major differences emerged from the analysis of the NG2-positive cells component of the cultures (Supplementary Figure S7A), while in Wt cultures the two highest concentrations produces a significant decrease of MBP-positive mature oligodendrocytes (One-Way ANOVA, $F(3,15) = 6.753$, $p = 0.0042$; Dunnet's post-test, 0.3 μM, $p = 0.0138$; 1 μM, $p = 0.0058$) (Supplementary Figure S7B). Representative images are included in Supplementary Figure S7C.

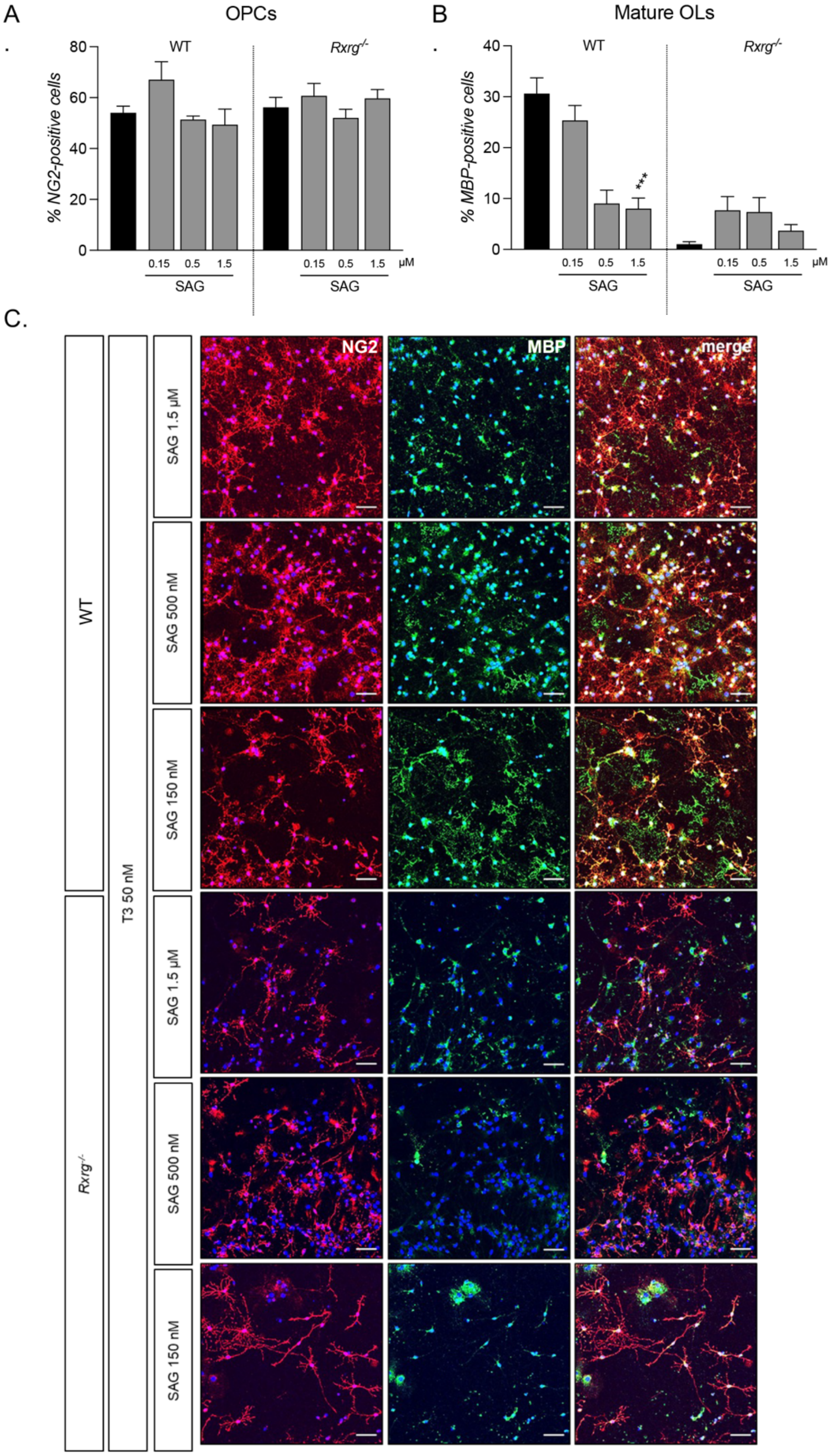
A
OPCs
WT
Rxrg-/-
% NG2-positive cells
80
60
40
20
0
0.15
0.5
1.5
μM
SAG
B
Mature OLs
% MBP-positive cells
40
30
20
10
***
C.
WT
Rxrg-/-
T3 50 nM
SAG 1.5 μM
SAG 500 nM
SAG 150 nM
NG2
MBP
merge

Figure Supplementary 6. Dose-response analysis of SHH agonist SAG.

(A - B) Graphs show the quantification of NG2-positive OPCs (A) and MBP-positive mature oligodendrocytes (B) treated with T3-only (black column) or together with SAG at different concentrations (gray columns) in Wt and Rxrg-/- cultures. (C) Representative images. Scale bar = 20 µm. Statistical analysis. One-Way Anova followed by Dunnett's t-test. Asterisks represent statistically significant differences in comparison with the T3-only treated group (black column) within the same genotype (*** $p < 0.001$).

Abbreviations: MBP, myelin basic protein; NG2, neuron-glial antigen2; OL, oligodendrocyte; OPC, oligodendrocyte precursor cell; SAG, smoothened agonist; T3, triiodothyronine; WT, wild type.

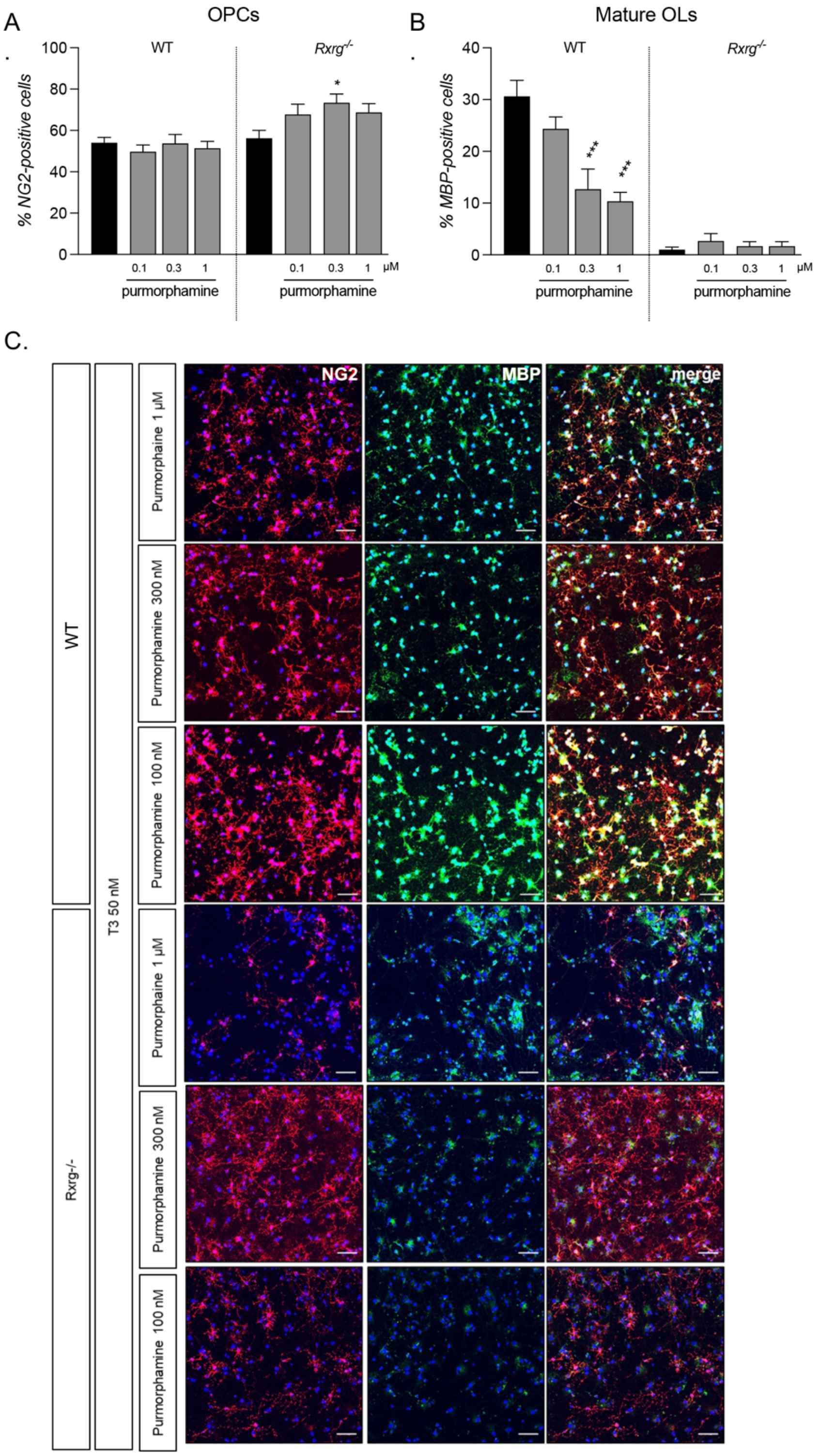
A
OPCs
% NG2-positive cells
100
80
60
40
20
0
WT
Rxrg-/-
*
0.1
0.3
1
µM
purmorphamine
purmorphamine
B
Mature OLs
% MBP-positive cells
40
30
20
10
0
WT
Rxrg-/-
***
***
0.1
0.3
1
µM
purmorphamine
purmorphamine
C.
NG2
MBP
merge
WT
Rxrg-/-
T3 50 nM
Purmorphaine 1 µM
Purmorphamine 300 nM
Purmorphamine 100 nM
Purmorphaine 1 µM
Purmorphamine 300 nM
Purmorphamine 100 nM

Figure supplementary 7. Dose-response analysis of SHH agonist purmorphamine.
(A - B) Graphs show the quantification of NG2-positive OPCs (A) and MBP-positive mature oligodendrocytes (B) treated with T3-only (black column) or together with purmorphamine at different concentrations (gray columns) in Wt and Rxrg-/- cultures. (C) Representative images. Scale bar = 20 µm. Statistical analysis. One-Way Anova followed by Dunnett's t-test. Asterisks represent statistically significant differences in comparison with the T3-only treated group (black column) within the same genotype (*** $p < 0.001$).
Abbreviations: MBP, myelin basic protein; NG2, neuron-glial antigen2; OL, oligodendrocyte; OPC, oligodendrocyte precursor cell; T3, triiodothyronine; WT, wild type.
